# The pursuit of happiness: A reinforcement learning perspective on habituation and comparisons

**DOI:** 10.1371/journal.pcbi.1010316

**Published:** 2022-08-04

**Authors:** Rachit Dubey, Thomas L. Griffiths, Peter Dayan

**Affiliations:** 1 Department of Computer Science, Princeton University, Princeton, New Jersey, United States of America; 2 Department of Psychology, Princeton University, Princeton, New Jersey, United States of America; 3 Max Planck Institute for Biological Cybernetics, Tübingen, Germany; 4 University of Tübingen, Tübingen, Germany; Peking University, CHINA

## Abstract

In evaluating our choices, we often suffer from two tragic relativities. First, when our lives change for the better, we rapidly habituate to the higher standard of living. Second, we cannot escape comparing ourselves to various relative standards. Habituation and comparisons can be very disruptive to decision-making and happiness, and till date, it remains a puzzle why they have come to be a part of cognition in the first place. Here, we present computational evidence that suggests that these features might play an important role in promoting adaptive behavior. Using the framework of reinforcement learning, we explore the benefit of employing a reward function that, in addition to the reward provided by the underlying task, also depends on prior expectations and relative comparisons. We find that while agents equipped with this reward function are less happy, they learn faster and significantly outperform standard reward-based agents in a wide range of environments. Specifically, we find that relative comparisons speed up learning by providing an exploration incentive to the agents, and prior expectations serve as a useful aid to comparisons, especially in sparsely-rewarded and non-stationary environments. Our simulations also reveal potential drawbacks of this reward function and show that agents perform sub-optimally when comparisons are left unchecked and when there are too many similar options. Together, our results help explain why we are prone to becoming trapped in a cycle of never-ending wants and desires, and may shed light on psychopathologies such as depression, materialism, and overconsumption.

## Author summary

Even in favorable circumstances, we often find it hard to remain happy with what we have. One might enjoy a newly bought car for a season, but over time it brings fewer positive feelings and one eventually begins dreaming of the next rewarding thing to pursue. Here, we present a series of computational simulations that suggest these presumable “flaws” might play an important role in promoting adaptive behavior. We explore the value of prior expectations and relative comparisons as a useful reward signal and find that across a wide range of environments, these features help an agent learn faster and adapt better to changes in the environment. Our simulations also highlight scenarios when these relative features can be harmful to decision-making and happiness. Together, our results help explain why we have the propensity to keep wanting more, even if it contributes to depression, materialism, and overconsumption.

## Introduction

*“Happiness is like a feather flying in the air. It flies light, but not for very long.”*

—Vinicius de Moraes, *A Felicidade*

From ancient religious texts to modern literature, human history abounds with tales describing the struggle to achieve ever-lasting happiness. Paradoxically, happiness is one of the most sought-after human emotions, yet achieving it over the long-term remains an elusive goal for many people. This pursuit turns out to be hard particularly because happiness is not just a function of one’s current state, but is plagued by two relativities. First, what it takes to be happy depends on one’s prior expectations, and these expectations adapt to current circumstances [1–4]. A positive change in lifestyle produces a “boost” in happiness but the boost often does not last long and one rapidly habituates to the higher standard of living (aptly coined the “hedonic treadmill” [5]). Second, happiness is influenced by relative comparisons [6–9]. Beyond the absolute level of what they have, people are often concerned with the difference between what they have and a desired level that they wish to achieve (the so-called “aspiration level” [10, 11]). The dynamics of adaptive expectations and relative comparisons have significant consequences for mental health and well-being—they can result in a vicious cycle of never-ending wants and desires, leaving us miserable even in favorable circumstances [12–16].

The conspicuous and turbulent impact of these features on society raises an important question: *why* is behavior influenced by habituation and comparison in the first place? A longstanding assumption within the field is that these relative features might have offered evolutionary advantages [17–21], however, a precise characterization of how and why these relative aspects might be desirable features of intelligent agents remains lacking.

In this article, we provide a perspective on the costs and benefits of habituation and comparisons by adopting the computational framework of reinforcement learning (RL) [22], within which we can make a formal distinction between what one might call objective and subjective reward functions. RL has historically offered a rich and comprehensive framework for understanding behaviors that are guided by value [23–26]. In standard RL theory, the reward function serves the role of *defining* optimal behavior i.e., what the agent “ought” to accomplish. This is easy in experimentally-determined environments, but harder in more open-ended natural environments [27–29]. Nevertheless, in either case, such reward functions are *objective* in the sense they are determined directly by the current task and they identify what an environment designer (or, in a much more complicated sense, evolution) desires the agent to achieve.

However, recently, the machine-learning literature has embraced the observation that the reward function plays a second, critical, role in RL in *steering* the agent from incompetence to mastery, and so has investigated strategies for *reward design* [30–35]. These steering reward functions, often provided by the designer to the agent, have subjective features detached from the particular task but can nevertheless guide the learning of the agent. Here, we consider whether subjective reward functions based on habituation and comparisons can steer agents competently towards objective success.

Specifically, we endow agents with a subjective reward function that, in addition to the task-specific objective reward, also depends on prior expectations and relative comparisons. We then embed these agents in various parameterized environments and compare their performance against standard RL agents whose reward function depends on just the objective reward value. Extensive computational simulations reveal that agents whose reward functions depend on these additional features learn faster and significantly outperform standard reward-based agents. Notably, this reward function offers substantial benefits in sparsely-rewarded and non-stationary environments, settings that are considerably challenging for standard reinforcement learning.

Our results suggest that a subjective reward function based on prior expectations and comparisons might play an important role in promoting adaptive behavior by serving as a powerful *learning* signal. This provides computational support for a longstanding assumption in the field and explains *why* the human reward function might be based on these features. At the same time, our simulations also shed light on potential pitfalls of this function. We find that agents perform sub-optimally in settings where aspiration-levels are left unchecked and become too high. We also find that these agents suffer from the “paradox of choice” [36]—when they are in a setting where different options are very similar to each other, they do not improve performance and remain constantly dissatisfied.

Taken together, our results provide a computational foundation for a reward function based on adaptive expectations and relative comparisons, and may shed light on psychopathologies such as depression, materialism, and overconsumption.

## Model

### Formalism: Reinforcement learning

To provide the foundation for our approach, we first review the formalism of reinforcement learning (RL) in the context of Markov decision problems (MDPs).

RL describes how an agent interacting with an environment can learn to choose its actions to maximize expected long-run future task reward. The environment includes distinguishable states (like a position in a maze), which can be identified with an observation that the agent makes, actions which lead to transitions between states (like moving in a cardinal direction), and objective rewards which are typically scalar (though can also be zero or negative and depend stochastically or deterministically on the state and/or action). That is, at each step *t*, the agent executes an action *a*_*t*_, transitions from state *s*_*t*_ to state *s*_*t*+1_ and receives a reward *r*_*t*_. The agent acquires and maintains a possibly probabilistic policy *π*(*s*), which specifies a systematic mapping of states to distributions over actions.

In the simplest case, the goal of the agent is to maximize expected return following time *t*, where the return *J*_*t*_ is the sum of the rewards:

$$\begin{array}{c} J_{t}=r_{t}+r_{t+1}+r_{t+2}+\ldots +r_{T}, \end{array}$$ (1)

where *T* is a final time step. The approach of maximizing expected return makes sense in situations when there is a natural notion of a final time step. However, in cases where the agent-environment interaction can go on continually without limit, the agent tries to select actions to instead maximize expected *discounted* return, where the return *G*_*t*_ is defined as:

$$\begin{array}{c} G_{t}=r_{t}+\gamma r_{t+1}+\gamma^{2}r_{t+2}+\ldots =\sum_{k=0}^{\infty }\gamma^{k}r_{t+k}, \end{array}$$ (2)

where *γ* ∈ [0, 1] is the discount factor, a parameter that determines the present value of future rewards.

The first distinction we make between objective and subjective RL problems is common in the literature. That is, the objective problem involves a fixed lifetime for the agent and maximization of the expected return *J*_*t*_. However, the agent does not know this lifetime and instead solves a subjective RL problem of maximizing the expected discounted return *G*_*t*_.

RL algorithms are often based on estimating the value function—a function that estimates how good it is for the agent to be in a given state. Denoted as *V*^*π*^(*s*), the value function is defined as the expected cumulative discounted reward of being in a state *s* and taking actions thereafter according to policy *π*:

$$\begin{array}{c} V^{\pi }\left(s\right)=E_{\pi }[G_{t}|s_{t}=s]. \end{array}$$ (3)

The value function can also be defined recursively as the sum of the immediate reward and the discounted value of the successor state as follows:

$$\begin{array}{c} V^{\pi }\left(s\right)=E_{\pi }[r_{t}+\gamma V^{\pi }\left(s_{t+1}\right)|s_{t}=s], \end{array}$$ (4)

where *r*_*t*_ is the reward received upon taking action *a*_*t*_ in state *s*_*t*_ and transitioning to state *s*_*t*+1_. Here, the expectation E takes into account the randomness associated with the action chosen, the successor state, and the subsequent reward.

Similar to the state-value function, we can also define the action-value function which tells us the value of the different actions that can be taken in each state. This value of an action, called the *Q*-value and denoted as *Q*^*π*^(*s*, *a*), is defined as the expected discounted future reward associated with taking an action *a*_*t*_ in state *s*_*t*_ and following policy *π* thereafter:

$$\begin{array}{c} \begin{array}{cc} Q^{\pi }\left(s,a\right) & =E_{\pi }[G_{t}|s_{t}=s,a_{t}=a]\\ & =E_{\pi }[r_{t}+\gamma V^{\pi }\left(s_{t+1}\right)|s_{t}=s,a_{t}=a]. \end{array} \end{array}$$ (5)

Here, the initial action is set and the expectation accounts for the randomness in state transitions and subsequent rewards (both of which are conditioned on the action chosen). Furthermore, the relationship between the value function and the action-value function is given as:

$$\begin{array}{c} V^{\pi }\left(s\right)=E_{\pi }[Q^{\pi }\left(s,a\right)], \end{array}$$ (6)

where the expectation takes into account the actions *a* specified by *π*(*s*). In an MDP, there is at least one deterministic policy that maximizes the expected discounted return simultaneously at all states. This is called the optimal policy, π*(*s*), and is associated with an optimal *Q*-value function defined as:

$$\begin{array}{c} Q^{*}\left(s,a\right)=\underset{\pi }{\text{max}}\;Q^{\pi }\left(s,a\right)\;\text{with}\;\pi^{*}\left(s\right)={\text{argmax}}_{a}Q^{*}\left(s,a\right) \end{array}$$ (7)

for all *s* ∈ *S* and *a* ∈ *A*(*s*). The problem of reinforcement learning is then reduced to estimating the action values as accurately as possible to maximize total expected discounted return (although note that policy gradient methods [37] do not formally require action values, rather only using them for variance reduction [38]).

For our purposes, we consider agents that learn the action values via the *Q*-learning algorithm [39]. According to the *Q*-learning algorithm, following a transition *s*_*t*_ → *s*_*t*+1_ initiated by action *a*_*t*_, a reward prediction error, *δ*_*t*_, is calculated and used to update the *Q*-values:

$$\begin{array}{c} \begin{array}{cc} & \delta_{t}=r_{t}+\gamma \;\underset{a\in A}{\text{max}}\;Q\left(s_{t+1},a\right)-Q\left(s_{t},a_{t}\right)\\ & Q\left(s_{t},a_{t}\right)\leftarrow Q\left(s_{t},a_{t}\right)+\alpha \delta_{t}, \end{array} \end{array}$$ (8)

where *α* ∈ [0, 1] is the learning-rate parameter that determines how quickly the agent learns from the new experience. By updating *Q*(*s*, *a*) in proportion to the reward prediction error, the action values gradually approximate the optimal action values.

When the *Q*-values converge to their true values, the agent can act greedily by choosing actions with the highest estimated action value. During learning, however, the agent faces a difficult trade-off between exploration and exploitation [40]. If the agent does not explore the space of possible actions well enough, then the action values will converge too slowly or get stuck at a suboptimal point. If the agent explores too much, then it will yield a lower cumulative reward. Furthermore, in one of the classes of environments we examine, the environment is non-stationary (the objective reward function changes over time). This means that continual exploration is required. The exigencies of exploration constitute a second, and more subtle, deviation between objective and subjective RL problems [41]. The objective problem requires optimizing the expected return *J*_*t*_ from the very first action the agent takes. The subjective RL problem involves learning a policy that makes good choices in *future* interactions with the environment, assuming that it is stationary.

In our experiments, the agents learn the action values using the *ϵ*-greedy policy—at each state *s*_*t*_, with probability 1 − *ϵ*, the agent chooses the action with the highest estimated *Q*-value and with probability *ϵ* it chooses an action uniformly at random. The *ϵ*-greedy algorithm presents a relatively simple yet effective method to balance exploration and exploitation. Another (relatively) simple and commonly used exploration strategy is the softmax strategy, where the actions are selected with probabilities proportional to their current values. We chose *ϵ*-greedy over softmax primarily for computational convenience—like the *ϵ*-greedy strategy, the softmax strategy also has one free parameter but tuning this parameter is generally considered to be harder than tuning the *ϵ* parameter [42]. However, in environments with very sparse or noisy rewards, these strategies perform poorly as random exploration in these environments rarely discovers rewarding states and such scenarios often require more sophisticated methods for exploration [43–46]. Later, we will see that part of the reason why relative comparison improves the agents’ performance is that it provides an additional exploration incentive to the agents and implicitly encourages them to try new actions.

The *Q*-learning algorithm is a model-free RL method. Model-free methods [39, 47, 48] learn the action values directly, by trial and error, without learning a “model” of the environment (i.e., without acquiring and using information about the reward function and the one-step state transition distribution). This is computationally efficient but also causes inflexibility: the absence of a model means that whenever the environment changes, the entire action value function needs to be re-learned as direct experience of changed rewards and transitions. In contrast, model-based methods [41, 49, 50] acquire the underlying model of the environment, and use this model to estimate the action values by iterative computation, analogous to planning steps to take in a maze. These methods are computationally expensive, but their advantage lies in their flexibility: local changes in the environment which result in local changes in the model can nevertheless allow the agent to make global changes to its policy (for instance, if a current path in the maze is blocked off). The issues for exploration and exploitation differ rather substantially between model-based and model-free methods. Model-based methods can have a sophisticated knowledge of their own uncertainty, but then face a catastrophically hard computational problem of using this information to explore and exploit with optimal efficiency, whereas model-free methods are more simply guided by the reward they receive. Here, we focus on model-free RL (by using the *Q*-learning algorithm) to study how the severe limitations that arise from its impoverished use of the reward signal (from the perspective of exploration and exploitation) can be ameliorated by prior expectations and relative comparisons.

### The optimal reward framework

Our approach is based on the optimal reward framework [30, 31], a reward design method which aims to design an appropriate reward function to optimize the behavior of an RL agent.

The optimal reward framework proposes a transformation of the reward function into two functions—the *objective* and the *subjective* reward function. The objective reward function outlines the task i.e., what the agent designer wishes the RL agent to achieve (e.g., evolution might desire an agent to maximize fitness). However, objective rewards are usually sparse and delayed relative to the actions necessary to garner them, making the task considerably hard for the agent to solve. While the agent designer is taken as being unable to change the actual task, they can still choose the reward function they provide to the agent. This reward function, often a parametric transformation of the objective reward function, is called the *subjective* reward function and it can potentially provide useful feedback to the agent. The agent designer then faces the task of figuring out what this reward function that determines the way the agent learns should be such that the agent comes to satisfy as best as possible the designer’s objective over a desired distribution of environments. To solve this problem, in our case, the designer performs a grid search over the space of possible parameter values for a parameterized subjective reward function and evaluates the expected cumulative objective reward in a single environment or a class of environments. This results in an (empirical) *optimal reward function* i.e., the subjective reward function that when used by an agent to learn, allows it to maximize expected objective reward over the distribution of environments. Crucially, this subjective reward function can be different from the objective reward function but it may still offer a significant advantage over learning based on just the objective rewards.

The optimal reward framework is useful because it answers the question of what makes a good reward function while maintaining the generality of RL. We here use it to explore when and why prior expectations and relative comparisons can potentially serve as useful (subjective) reward functions.

### Approach

We consider an agent designer who seeks an RL agent to maximize cumulative objective reward, *J*_*t*_, over its finite lifetime. The designer then faces the task of choosing an appropriate subjective reward function with which to endow the agent, where each possible reward function is a weighted combination of three components:

$$\begin{array}{c} \begin{array}{cc} & f=w_{1}.\text{Objective}+w_{2}.\text{Expect}+w_{3}.\text{Compare},\\ & w_{1},w_{2},w_{3}\in \left[0,1\right]. \end{array} \end{array}$$ (9)

Here, the first component is Objective = *r*_*t*_, and it is the reward that the agent receives upon taking the action *a*_*t*_ in state *s*_*t*_ and transitioning to state *s*_*t*+1_. This component is the standard reward function used in the RL framework. The second component, Expect is intended to capture a reward function that is determined by prior expectations. We define this component as Expect = *r*_*t*_ − *Q*(*s*_*t*_, *a*_*t*_), where *r*_*t*_ is the objective reward received after taking the action *a*_*t*_ in state *s*_*t*_ and *Q*(*s*_*t*_, *a*_*t*_) is the previous action value estimate of that state-action pair. This component decreases the objective reward value according to the current expectation by subtracting the expected value *Q*(*s*_*t*_, *a*_*t*_) from *r*_*t*_. Further, this component is adaptive—during learning, *Q*(*s*_*t*_, *a*_*t*_) keeps getting updated and thus the agent’s expectation will adapt to new experiences. Note that this component is quite similar to the reward prediction error *δ*, as defined in Eq 8. One difference, however, is that we set the *γ* value of the Expect component to be 0 to focus on local prediction errors and myopic expectations, which makes Expect ultimately negative in many environments. The third component Compare, is given as Compare = *r*_*t*_ − *ρ*, where *ρ* is an aspiration level for the agent. At each step *t*, this component evaluates the reward received by the agent, *r*_*t*_, in comparison to the aspiration level thereby capturing a reward function that is determined by relative comparisons. The aspiration level could presumably be learnt by an agent via peer comparisons or it could be inherited directly from culture or parents. Here, we assume that the designer directly provides the aspiration level to the agent. Thus, in addition to providing the subjective reward, the designer also faces the challenge of providing an appropriate aspiration level to the agent.

In detail, we fix the agents to be *ϵ*-greedy tabular *Q*-learning agents where the *Q*-update is performed over the subjective reward function that is chosen by the agent designer. Following algebraic manipulations, the learning rule for the agents can be written as below:

$$\begin{array}{c} \begin{array}{cc} & f=\left(w_{1}+w_{2}+w_{3}\right).r_{t}-w_{3}.\rho -w_{2}.Q\left(s_{t},a_{t}\right),\\ & \delta =f+\gamma \;\underset{a\in A}{\text{max}}\;Q\left(s_{t+1},a\right)-Q\left(s_{t},a_{t}\right),\\ & Q\left(s_{t},a_{t}\right)\leftarrow Q\left(s_{t},a_{t}\right)+\alpha \delta . \end{array} \end{array}$$ (10)

Here, in contrast to the standard *Q*-learning algorithm as defined in Eq 8, the reward function *f* is not just dependent on the objective reward value of a state-action pair *r*_*t*_, but it can also be influenced by prior expectation and relative comparison (given that the designer assigns these weights to be different from zero). As a consequence, the *Q*-value estimate following Eq 10 will differ substantially from the standard *Q*-learning algorithm, which will eventually impact how these agents learn and act in the environment.

As noted, the main aim of the paper is to study how prior expectations and relative comparisons can impact learning. That said, the idea is around that a function similar to Eq 9 can model some aspects of an individual’s well-being [51, 52], acknowledging, of course, that this is simplistic compared with the careful psychological and neural analyses dissociating hedonic and motivational aspects of reward [53, 54], and given other factors influencing happiness. Nevertheless, in order to explore how some of our simulation results might at least be somewhat analogous to psychological findings in this domain, we explore the consequences of assuming that the (momentary) happiness level of the agent is determined by the subjective reward value that the agent receives after taking an action. Thus, when *w*_2_ ≠ 0 and *w*_3_ ≠ 0, the agent’s happiness is influenced by prior expectations and relative comparisons, and when *w*_2_ = *w*_3_ = 0, the agent’s happiness is only influenced by the objective reward value.

We now present various computational experiments in which we vary the distribution of environments, while keeping the agents and the objective reward function fixed, and derive the optimal reward function for each setting. To find the optimal reward function, we perform a (nearly) exhaustive search over the weights *w*_1_, *w*_2_, and *w*_3_ and the aspiration level, *ρ*, and compare the average cumulative objective reward of the different agents. Further, we also use grid search to find approximately optimal, but fixed, values of other parameters such as the learning rate *α* and the exploration parameter *ϵ* (see Methods). Because we specify the objective function to correspond directly to the first component of *f*, any set of environments that results in an optimal reward function where the values of *w*_2_ and *w*_3_ are not equal to zero can supply insights about when and why prior expectations and relative comparisons are useful reward signals.

We expect the optimal subjective reward function to depend sensitively on the environments with which agents are faced. We therefore consider a range of paradigmatic challenges. Facets we consider include the density and stationarity of rewards and the recurrence of the domain; we also study the structurally simpler domain of bandit problems to illuminate our findings further.

## Results

### Experiment 1: Exploring the value of prior expectations and relative comparisons

#### Environment design

**Fig 1:**
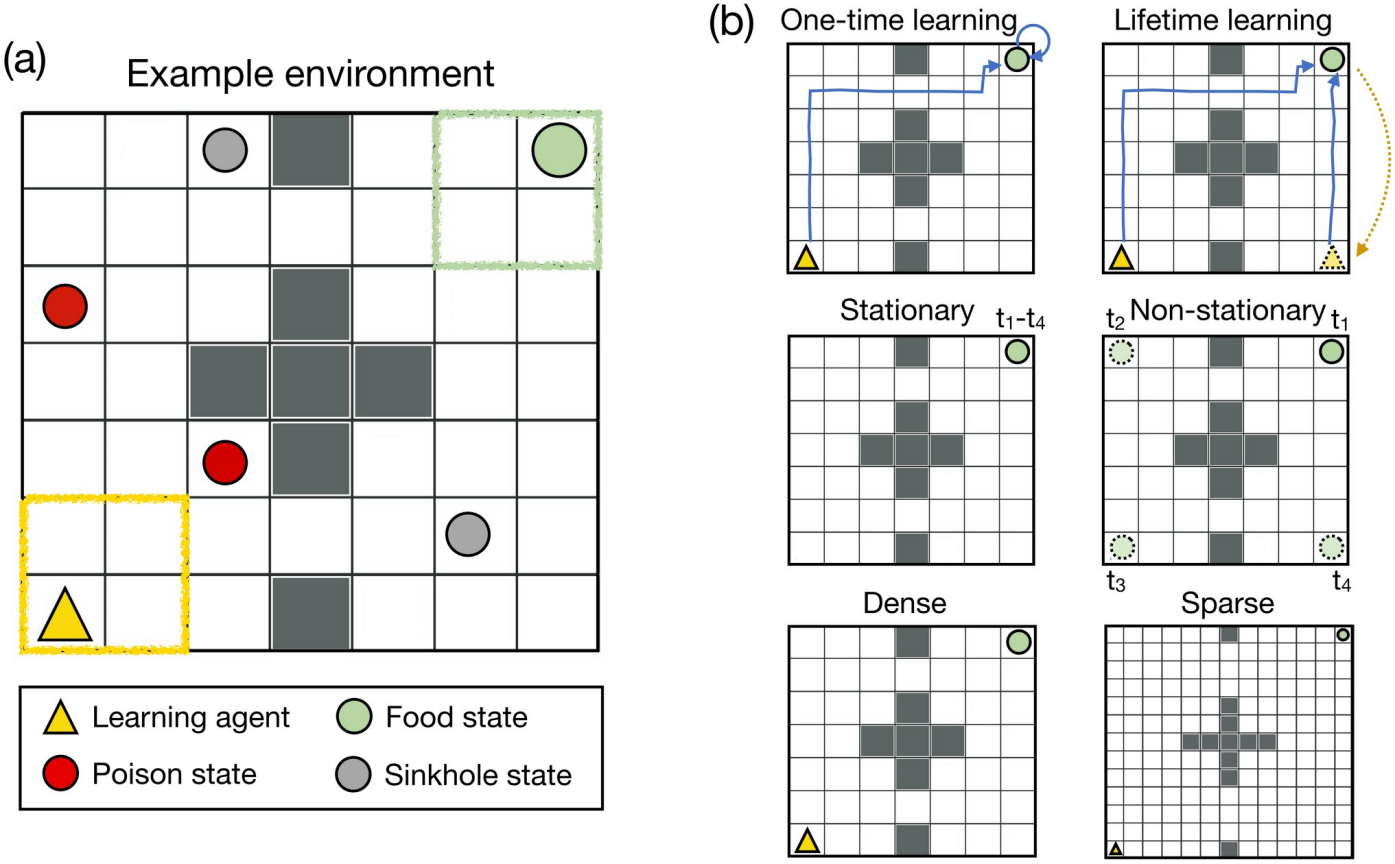
Environment design. **(a)** The two-dimensional gridworld environment used in Experiment 1. **(b)** To study the properties of the optimal reward, we made several modifications to the gridworld environment. Top row: In the *one-time* learning environment, the agent could chose to stay in the food location constantly after reaching it. In the *lifetime* learning environment, the agent was teleported to a random location in the gridworld as soon as it reached the food state. Middle row: In the *stationary* environment, the food remained in the same location throughout the agent’s lifetime. In the *non-stationary* environment, the food changed its location during the agent’s lifetime. Bottom row: We used a gridworld of size 7 × 7 to simulate a *dense* reward setting. To simulate a *sparse* reward setting, we increased the size of the gridworld to 13 × 13.

For our first set of experiments, we use a simulated physical space shown by the gridworld environment in Fig 1a. The gridworld environment is a popular testbed for various RL studies [22, 31, 55, 56] and lays out a straightforward way to study and model sequential decision-making. A single agent resides in the gridworld, and can choose between five actions: Up, Down, Right, Left, and Stay. Upon taking an action, with 90% probability the agent moves one step in the direction of the intended action and with 10% probability it randomly adopts one of the four other actions. The thick dark grey lines in the figure represent obstacles (walls) that the agent can not cross (regardless of the movement action), so it has to navigate through gaps in the walls to move to adjacent subspaces. At the beginning of training, one location (randomly picked between the four states in the top-right area of the gridworld; possible locations shown in the green box in the figure) contains the food, and the agent receives an objective reward of +1 whenever it is in the food state. For generality, we also include poison and sinkhole states, states which the agent should learn to avoid, in random locations in the environment (2 each in environments of size 7 × 7 and 4 each in environments of size 13 × 13). The agent receives an objective reward of −1 at the poison states and while the agent receives an objective reward of 0 at sinkhole states, they are very hard to get out of (the agent stays in the state with 95% probability regardless of the chosen action). While our motivation to include these states was to study avoidance behavior, we note that our qualitative results do not depend on the number of poison and sinkhole states we include in the environment. We also refer the reader to the S1 Text where we replicate our results in environments that contained no poison and sinkhole states. The agent receives an objective reward of 0 at all the other states. The agent’s starting location is one of the four states in the bottom-left area of the gridworld, opposite the food quadrant (possible locations shown in the yellow box in the figure).

A significant advantage of using the gridworld environment is that we can easily modulate it to make the task harder/easier for the agent to solve (also refer to Fig 1b). The simplest environment we use is a stationary gridworld of size 7 × 7, where the optimal policy takes 12 steps on average to reach the food state from the start state. Further, in this environment, once the agent reaches the food state, it can choose to stay in the food location constantly and keep accumulating objective rewards (Exp 1a, first simulation). Thus, this environment essentially only requires *one-time* learning.

We then modify the environment such that the agent teleports to a random location in the gridworld as soon as it reaches the food state. This environment requires *lifetime learning* as the agent has to learn how to reach the food state from any random state in the environment (Exp 1a, second simulation). After this, we increase the difficulty significantly via two important modifications. In Exp 1b, we modify the environment to make it *non-stationary* such that the food changes its location during the agent’s lifetime. This means that the agent has to re-learn the optimal policy whenever the environment changes. In Exp 1c, we modulate the difficulty by increasing the size of the environment from 7 × 7 to 13 × 13. This doubles the number of steps the optimal policy takes to reach the food state and simulates a *sparser* reward setting. Both flexible behavioral change and learning via delayed rewards are important aspects of intelligent behavior and these settings often provide significant challenges to standard model-free RL [22, 44, 48, 57, 58]. In simulating these environments we can study whether prior expectations and comparisons might help overcome these challenges.

#### Exp 1a: Comparison provides an exploration incentive and improves learning significantly

We begin by simulating a simple 7 × 7 stationary environment that requires one-time learning. Ideally, the agent should find the food state as quickly as possible and then it should stay in that state for the rest of the lifetime. To evaluate how well the different reward functions fulfill the designer’s objective, we compare the average cumulative objective reward at the end of the respective agents’ lifetime.

The reward functions we consider can be classified into seven different categories (refer to Table 1). For all analyses that follow, we report the best performing agent (along with the corresponding parameter values) within each of these reward function categories.

**Table 1:** Categories of reward functions. The reward functions we consider can be classified into seven categories. First is ‘Objective only’, where the reward function depends only on the first component, *w*_1_. Similarly, ‘Expect only’ is the function that depends only on the second component, *w*_2_, and ‘Compare only’ is the function that depends solely on the third component, *w*_3_. Then, we have the functions that are a combination of two components—‘Objective+Expect’, ‘Objective+Compare’, and ‘Expect+Compare’. Finally, we have the reward function, ‘All’, that depends on all three components.

Reward function category	*w*_1_ value	*w*_2_ value	*w*_3_ value
‘Objective only’	> 0	= 0	= 0
‘Expect only’	= 0	> 0	= 0
‘Compare only’	= 0	= 0	> 0
‘Objective+Expect’	> 0	> 0	= 0
‘Objective+Compare’	> 0	= 0	> 0
‘Expect+Compare’	= 0	> 0	> 0
‘All’	> 0	> 0	> 0

**Fig 2:**
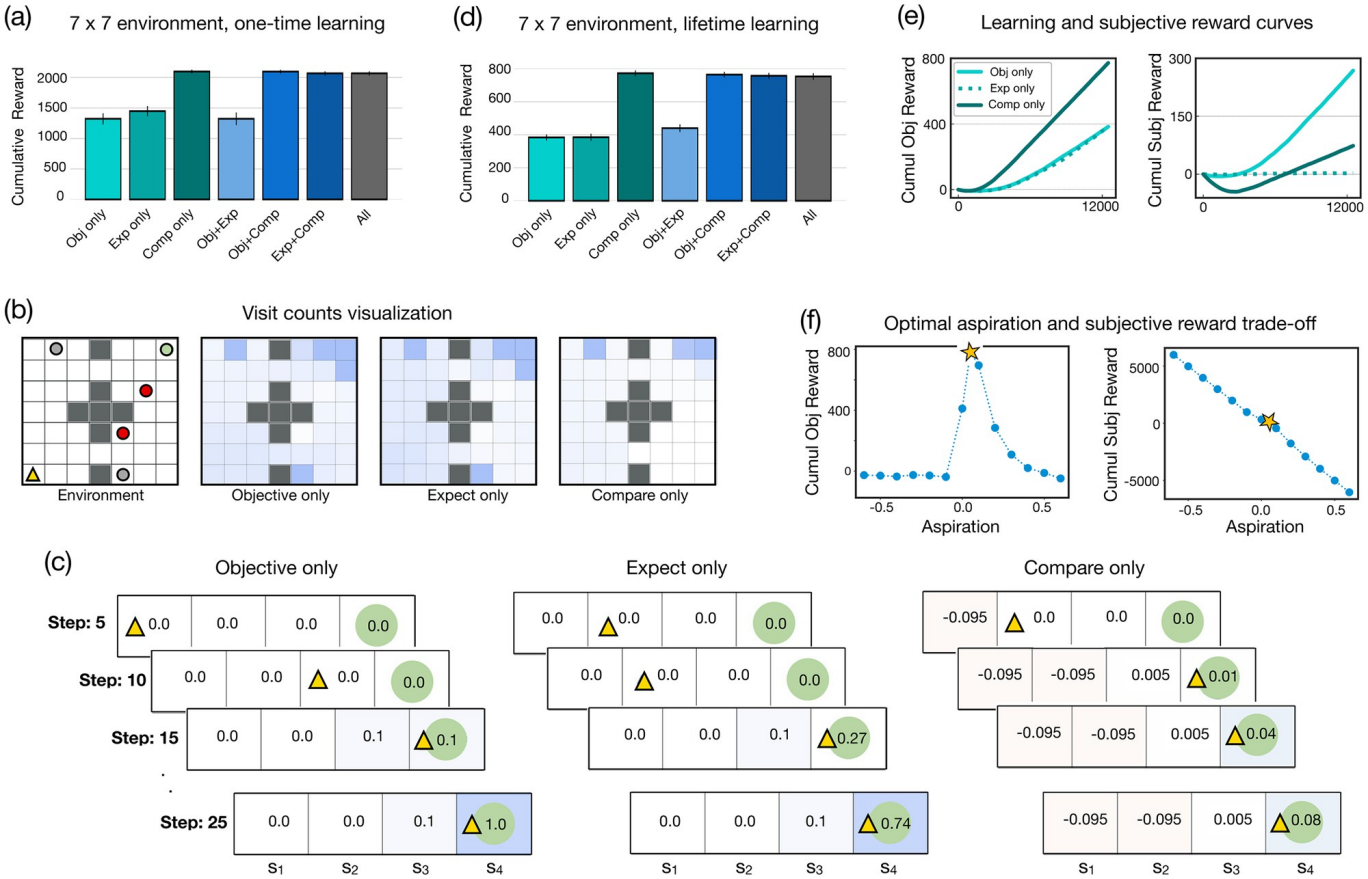
Comparison improves learning in simple dense, stationary environments. **(a)** Mean cumulative objective reward attained by the different agents in a distribution of 7 × 7 stationary environments requiring one-time learning (lifetime = 2500 steps). Here, relative comparison significantly improve performance and the ‘Compare only’ agent obtains the highest cumulative objective reward. **(b)** Average visit counts of the ‘Objective only’, ‘Expect only’, and ‘Compare only’ agents (darker color represents higher visit counts and vice-versa). Compared to the ‘Objective only’ and the ‘Expect only’ agents, the ‘Compare only’ agent spends very little time visiting the non-food states in the world. **(c)** Simulation of the agents’ behavior in a simple 4-state environment. The ‘Compare only’ agent assigns a negative value to any non-food state it visits (due to its aspiration level) which encourages it to visit novel states in the environment. This allows the agent to find the food location very quickly. The ‘Objective only’ and ‘Expect only’ agents primarily rely on random exploration and find the food location more slowly (*α* = 0.1 for all agents). **(d)** Mean cumulative objective reward attained by the different agents in a distribution of 7 × 7 stationary environments, requiring lifetime learning (lifetime = 12500 steps). The ‘Compare only’ agent again obtains the highest cumulative objective reward. **(e)** Time course of the cumulative objective reward attained by the different agents in the 7 × 7 environment requiring lifetime learning (left) and the time course of cumulative subjective reward experienced by the different agents (right). **(f)** Left: Mean cumulative objective reward attained by the ‘Compare only’ agent as a function of its aspiration level in the lifetime learning environment. The performance of the agent drops if the aspiration level is set to be too high or too low (the optimal aspiration level is marked in yellow). Right: Mean cumulative subjective reward of the agent as a function of its aspiration level (optimal aspiration level agent is shown in yellow marker).

Fig 2a plots the mean cumulative objective reward of the best agents from each reward function category (*α* = 0.9, *ϵ* = 0.01 for all agents). We find that the ‘Compare only’ agent (*w*_3_ = 0.4, *ρ* = 0.9) obtains the highest cumulative objective reward (*M* = 2097.02, *SD* = 219.35), more than the standard reward-based agent, ‘Objective only’ (*M* = 1321.89, *SD* = 644.61; *w*_1_ = 0.3), as well as the expectation-based agent, ‘Expect only’ (*M* = 1447.47, *SD* = 588.75; *w*_2_ = 0.8). Further, the ‘Compare only’ agent outperforms the ‘Objective+Expect’ agent (*M* = 1322.42, *SD* = 733.86; *w*_1_ = 1.0, *w*_2_ = 1.0), and performs equivalently to the ‘Objective+Compare’ agent (*M* = 2095.44, *SD* = 219.97; *w*_1_ = 0.4, *w*_3_ = 0.4, *ρ* = 0.9), the ‘Expect+Compare’ agent (*M* = 2067.29, *SD* = 242.58; *w*_2_ = 0.1, *w*_3_ = 0.8, *ρ* = 0.9), and the ‘All’ agent (*M* = 2066.72, *SD* = 242.62; *w*_1_ = 0.7, *w*_2_ = 0.4, *w*_3_ = 0.8, *ρ* = 0.9).

To provide some intuition about why the ‘Compare only’ agent performs so well, we simulate the agents’ behavior in a simple 4-state environment. This environment contains no obstacles or poison and sinkhole states, and the agent always moves with 100% probability in the direction of the intended action. The agent has three movement actions: Right, Left, and Stay, and its starting location is state *s*_1_ and the food location is in state *s*_4_. As in the main gridworld experiment, all non-food states provide an objective reward of 0 and the food state provides an objective reward of +1. The agent can again stay at the food location constantly, thereby requiring one-time learning. Fig 2(c) shows a visual representation of one instance of the agent-environment interaction history along with the subsequent learnt state values for the ‘Objective only’, ‘Expect only’, and ‘Compare only’ agents. We see that near the beginning of its lifetime (step = 5), the ‘Compare only’ agent assigns a negative value to *s*_1_. This is because this state gives an objective reward of 0 but the agent’s aspiration level is higher than this value (= 0.95) and thus, the agent receives a subjective reward of −0.95 at this state. This encourages the agent to move to *s*_2_ as the current value of *s*_2_ is 0 and greater than the value of *s*_1_. After reaching *s*_2_, the agent again assigns a negative value to *s*_2_ and then moves to *s*_3_ and again assigns it a negative value, and then moves to *s*_4_ where the food is located. Upon reaching the food state, the agent receives a positive subjective reward of 0.05, *r*_4_ = 1 and *ρ* = 0.95, which encourages it to stay in the food location for the remainder of its lifetime. In contrast, in the absence of exploration bonuses such as novelty bonus, optimistic initialization etc., the ‘Objective only’ and ‘Expect only’ agents assign a value of 0 to both *s*_1_ and *s*_2_ at the beginning of their lifetime (since they both receive a subjective reward of 0 at these states). Consequently, at *s*_2_, the agents are equally likely to move to *s*_1_ as they are to move to *s*_3_ (since the value of all these states is 0). As a result, these agents take a longer time to eventually reach the food location. In sum, relative comparison helps learning because it provides an *exploration incentive*—the agent quickly comes to assign a negative value to any non-food state it visits (due to its aspiration level) which indirectly encourages the agent to visit novel states.

We suggest that it is this difference in the way these agents learn and explore that helps the ‘Compare only’ agent to obtain higher cumulative objective reward in the larger and more complex gridworld environment. To illustrate this, Fig 2b plots the average number of times the agents visit the different states of the gridworld during their lifetime. We observe that in contrast to the ‘Objective only’ and the ‘Expect only’ agent, the ‘Compare only’ agent spends less time visiting the non-food states in the world. The ‘Compare only’ agent avoids the non-food states it has already visited (as the value of those states is negative) and it instead prefers to visit the states it has not yet visited (as the value of those states is zero), allowing it to find the food location very quickly. On the other hand, until the time that the ‘Objective only’ and ‘Expect only’ agents first visit the food state, they assign a value of zero to all non-food states regardless of their visit counts (except the poison state which issues a negative objective reward = −1). Thus, these agents are equally likely to move to a previously visited state as they are to move to a novel state which results in them finding the food location more slowly (as they primarily rely on random exploration to find the food location).

To complete our analysis of the dense, stationary environment, our next simulation considers a 7 × 7 environment that requires lifetime learning, where the agent is teleported to a random location in the world as soon as it reaches the food state (*α* = 0.5, *ϵ* = 0.1 for all agents). We find that in this setting (Fig 2b), the ‘Compare only’ agent (*w*_3_ = 0.5, *ρ* = 0.05) again accumulates the highest cumulative objective reward (*M* = 771.84, *SD* = 180.48) greater than the ‘Objective only’ (*M* = 383.38, *SD* = 184.67; *w*_1_ = 0.7) and the ‘Expect only’ agent (*M* = 384.68, *SD* = 215.28; *w*_2_ = 0.1). Further, the ‘Compare only’ agent performs equivalently to the ‘Objective+Compare’ agent (*M* = 763.51, *SD* = 169.32; *w*_1_ = 0.1, *w*_3_ = 0.8, *ρ* = 0.05), ‘Expect+Compare’ agent (*M* = 756.56, *SD* = 179.07; *w*_2_ = 0.1, *w*_3_ = 0.8, *ρ* = 0.01), as well as the ‘All’ agent (*M* = 752.45, *SD* = 205.88; *w*_1_ = 0.7, *w*_2_ = 0.1, *w*_3_ = 0.5, *ρ* = 0.01). Fig 2e (left plot) further demonstrates the learning difference between the ‘Objective only, ‘Expect only, and the ‘Compare only’ agents. The ‘Compare only’ agent learns faster and attains higher cumulative objective reward compared to the other agents. Fig 2e (right plot) shows the difference in the subjective rewards of the three agents throughout their lifetime. The subjective reward of the ‘Objective only’ agent is, naturally, proportional to the objective reward it receives in its lifetime. In some sense, the ‘Objective only’ agent is experiencing happiness in proportion to the objective reward it receives. The ‘Expect only’ agent, apart from small boosts in happiness (which occur on the first few food state visits), maintains a steady state of happiness throughout its lifetime, which is akin to the hedonic treadmill. The ‘Compare only’ agent experiences negative subjective reward in the early stages of training i.e., it can be thought as being more unhappy in the beginning (because of the initial visits to the non-food states). However, this then provides an exploration incentive to the agent and it visits the food state more regularly which eventually leads to higher subjective reward i.e., in some sense, its happiness rises after it learns a good policy. Taken together, these simulations suggest that given a distribution of dense, stationary environments (either requiring one-time or lifetime learning), a reward function based on comparison to a well chosen aspiration level optimizes the course of learning.

#### Optimal aspiration level and the trade-off between objective and subjective rewards

While the above results show that comparison serves as a useful learning signal, it is important to note that the aspiration level of comparison-based agents needs to be set appropriately in order for the agents to act optimally. Fig 2f plots the average cumulative objective reward obtained by the ‘Compare only’ agent as a function of the aspiration level in the dense, stationary, lifetime learning environment and shows that the performance of the agent is lowered if the aspiration level is set to be too high or too low. If the aspiration level is set to be too high, then the agent assigns high negative values to all the states it visits (since the subjective reward received at the states is very negative). This can cause the agent to become pessimistic in its exploration strategy and learn a sub-optimal policy. On the other hand, if the agent’s aspiration level is too low, then it learns more slowly as it is not encouraged to explore novel states.

We can also study the relationship between the aspiration level and the experienced subjective rewards of an agent. Agents that have very high aspiration level accumulate high negative subjective rewards in their lifetime (Fig 2f). Conversely, agents with very low aspiration level end up accumulating high positive subjective rewards in their lifetime. However, both these kinds of agents are not well-calibrated to the statistics of the environment. For example, an agent that has an aspiration level = −0.1 will be deluded as it would keep visiting states that provide an objective reward of 0 (since they give subjective reward = 0.1) and will most likely never discover the food location. Thus, agents that experience too many positive subjective rewards or too many negative subjective rewards do not obtain high objective rewards. In some sense, this perhaps suggests that both being too happy or too unhappy results in poor performance and agents that obtain the highest cumulative objective reward tend to experience a moderate amount of unhappiness in their lifetime. While our definition of happiness is obviously very simple, this analysis acts as a demonstration exercise to show the trade-off an agent designer faces in terms of maximizing the subjective reward accumulated by an agent and the cumulative objective reward accrued by that agent.

#### Exp 1b: Prior expectation and comparison help deal with non-stationarity

For our next simulation, we study the properties of the optimal reward function in a non-stationary environment. As before, at the beginning of the agent’s lifetime, the food is randomly located in one of the four states in the top-right area of the 7 × 7 gridworld. Once the agent reaches the food location, it can choose to stay there constantly (i.e., it is not teleported to a random location in the world). However, every 1250 steps, the food changes its location and moves to one of the other remaining corners of the gridworld requiring the agent to continue exploring in order to find the new food location.

**Fig 3:**
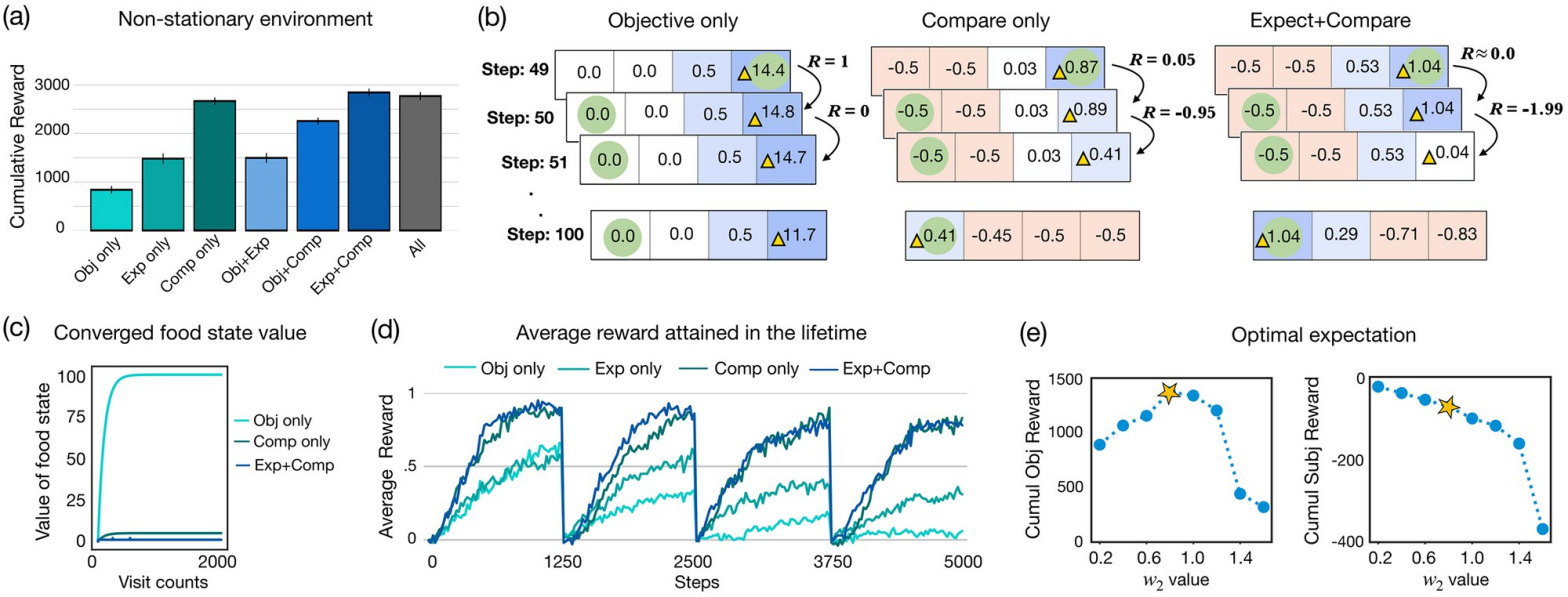
Prior expectation and comparison make an agent robust to changes in the environment. **(a)** Mean cumulative objective reward attained by the agents in a distribution of 7 × 7 non-stationary environments (lifetime = 5000 steps). Both prior expectation and relative comparisons are helpful in dealing with non-stationarity. **(b)** Agents’ behavior in a simple 4-state non-stationary environment. By step = 50, the ‘Objective only’ agent assigns a considerably higher value to the food state compared to the ‘Compare only’ and ‘Expect+Compare’ agents. At step = 51, when the food changes its location, the ‘Objective only’ agent receives a subjective reward of 0 at state *s*_4_ and takes a long time to lower the value of this state. Even by step = 100, it is not able to discover the new food location. In contrast, after the food changes location, the ‘Compare only’ and ‘Expect+Compare’ agents receive high negative subjective rewards at state *s*_4_ which reduces their value estimate of *s*_4_ very quickly. This encourages them to visit other states and enables them to discover the new food location very quickly. **(c)** Graph showing how the value of the food state changes as a function of the visit counts for the different agents. While the state value converges for all the three agents, the ‘Objective only’ agent ends up assigning a very high value to the food state because it receives a subjective reward = 1 at each visit. The ‘Compare only’ and ‘Expect only’ agents receive lower subjective rewards and hence the converged state value is considerably lower for these agents. **(d)** Average reward rate of the various agents during their lifetime on the 7 × 7 gridworld environment (the food changes its location after every 1250 steps). **(e)** Left: Mean cumulative objective reward attained by the ‘Expect only’ agent as a function of the *w*_2_ values in the non-stationary environment. The performance of the agent drops if the weight is too high or too low (optimal *w*_2_ value is marked in yellow). Right: Mean cumulative subjective reward of the ‘Expect only’ agent as a function of the *w*_2_ value.

Fig 3a plots the mean cumulative objective reward of the best performing agents from each reward category. Here, the ‘Objective only’ agent (*w*_1_ = 0.4, *α* = dynamic, *ϵ* = 0.1) performs very poorly, obtaining the lowest objective reward (*M* = 840.10, *SD* = 578.24). It is outperformed by both the ‘Expect only’ (*M* = 1483.05, *SD* = 766.75; *w*_2_ = 0.9, *α* = 0.9, *ϵ* = 0.1), and the ‘Compare only’ agent (*M* = 2669.08, *SD* = 539.81; *w*_3_ = 0.2, *ρ* = 0.9, *α* = 0.1, *ϵ* = 0.1). While the ‘Compare only’ agent outperforms the ‘Expect only’ agent, we find that the ‘Expect+Compare’ agent (*M* = 2846.06, *SD* = 587.77; *w*_2_ = 0.1, *w*_3_ = 0.6, *ρ* = 0.9, *α* = dynamic, *ϵ* = 0.1) performs better than the ‘Compare only’ agent thereby suggesting that both prior expectation and comparison are helpful in dealing with non-stationarity.

To gain an intuition about why these factors help in non-stationary environments, we again look at the agent’s behavior in the previously described simple 4-state environment. As before, the agent’s starting location is *s*_1_ and the food is located at *s*_4_. To simulate non-stationarity, at step = 50, the food changes its location from *s*_4_ to *s*_1_ and it stays there till the end of the agent’s lifetime (lifetime = 100). Fig 3b shows a visual representation of one instance of the agent-environment interaction history (starting from step = 49) for the ‘Objective only’, ‘Compare only’, and ‘Expect+Compare’ agents. We first see that the agents differ in the way they update the value of a rewarding state as a function of the number of times they visit that state. For example, at step = 50, the ‘Objective only’ agent’s state value estimate for *s*_4_ is considerably higher than the ‘Compare only’ and ‘Expect+Compare’ agents’ estimate. This is because the ‘Objective only’ agent receives a subjective reward of +1 each time it visits the food state whereas the ‘Compare only’ agent only receives a subjective reward of 0.05 (*ρ* = 0.95). The ‘Expect+Compare’ agent receives a subjective reward = 1.05 at the first visit but it then receives a subjective reward close to 0 for subsequent visits. More generally, the *converged* state value estimate for the food state differs substantially for the three agents. As shown in Fig 3c, given *γ* = 0.99, the state value estimate of the food state converges to 100 for the ‘Objective only’ agent whereas it converges to 5 for the ‘Compare only’ agent, and to 1.04 for the ‘Expect+Compare’ agent (also refer to the S1 Text for a derivation of the convergence and the respective upper and lower bound for the different agents).

At step = 50, the food location changes from *s*_4_ to *s*_1_ but all three agents stay at *s*_4_ because the estimated value of *s*_4_ is higher than the estimated value of *s*_3_. The ‘Objective only’ agent then receives a subjective reward = 0 which reduces the agent’s estimated value of *s*_4_. However, because the previous estimated value of *s*_4_ is so high, it takes a long time for the value of *s*_4_ to become lower than *s*_3_ which results in the agent remaining in *s*_4_ for a long period. The ‘Compare only’ agent receives a high negative subjective reward upon visiting *s*_4_ (= −0.95) and because the value estimate of *s*_4_ is not very high, the value estimate reduces very quickly prompting the agent to explore new locations and eventually find the new food location at *s*_1_. The ‘Expect+Compare’ agent receives an even higher negative subjective reward (subjective reward induced by the Expect component = −1.04 and the Compare component = −0.95) and thus, the agent’s value estimate of *s*_4_ reduces even more quickly and it ends up finding the new food location faster than all other agents.

The simulation results for the 4-state environment are consistent with the behavior of the agents in the 7 × 7 gridworld experiment. Fig 3d plots the average reward rate of the ‘Objective only’, ‘Expect only’, ‘Compare only’, and ‘Expect+Compare’ agents during their lifetimes on the gridworld environment. Here, the food changes its location every 1250^*th*^ step and the ‘Objective only’ agent is not able to learn and discover the new location of the food. The ‘Expect only’ is able to better deal with the change in the environment as it is eventually able to find the new location of the food. The ‘Compare only’ agent also handles the change in the environment very well and it comfortably outperforms the ‘Expect only’ agent primarily because it is more efficient in its exploration (see also the previous section). Finally, the ‘Expect+Compare’ agent boosts the performance of the ‘Compare only’ agent as it is able to find the new food location faster. These results suggest that in non-stationary environments, both prior expectations and relative comparisons are valuable components as they help an agent quickly ‘move on’ from states that used to be rewarding in the past.

#### Optimal expectations

Similar to the relationship between the aspiration level and the subjective reward experienced by the comparison-based agents, the experienced subjective reward and performance of the ‘Expect only’ agent also depend considerably on the value of *w*_2_ (especially in the non-stationary environment). Fig 3e(left) plots the mean cumulative objective reward obtained by the ‘Expect only’ agent in the non-stationary environment as a function of *w*_2_, showing that performance is optimized at an intermediate point when the prior expectation is neither too high nor too low. By contrast with this non-monotonicity, Fig 3e(right) shows that agents with very low expectations (low *w*_2_) obtain high subjective reward in their lifetime despite their lack of high cumulative objective reward, whereas agents that have high expectations (by having high *w*_2_) obtain high negative subjective reward and still without attaining high objective reward. In some sense, this suggests that agents with low expectations are very happy in their lifetimes without performing well and agents with very high expectations are very unhappy while also not performing well. On the other hand, agents with moderate expectations tend to obtain the highest cumulative objective reward while experiencing some amount of unhappiness in their lifetime. This further demonstrates the trade-off an agent designer faces with regards to maximizing the cumulative objective reward and the happiness experienced by an agent. Note that the agent designer does not face this trade-off for the ‘Objective only’ agent—the happiness of which is directly proportional to its objective reward. Thus, the ‘Objective only’ agent that accrues the highest cumulative objective reward is the happiest such agent.

#### Exp 1c: Reward sparsity requires controlling comparisons

We now study the properties of the optimal reward function in a sparser reward environment using a 13 × 13 gridworld that requires *lifetime* learning, where the agent is teleported to a random location in the environment as soon as it reaches the food state.

**Fig 4:**
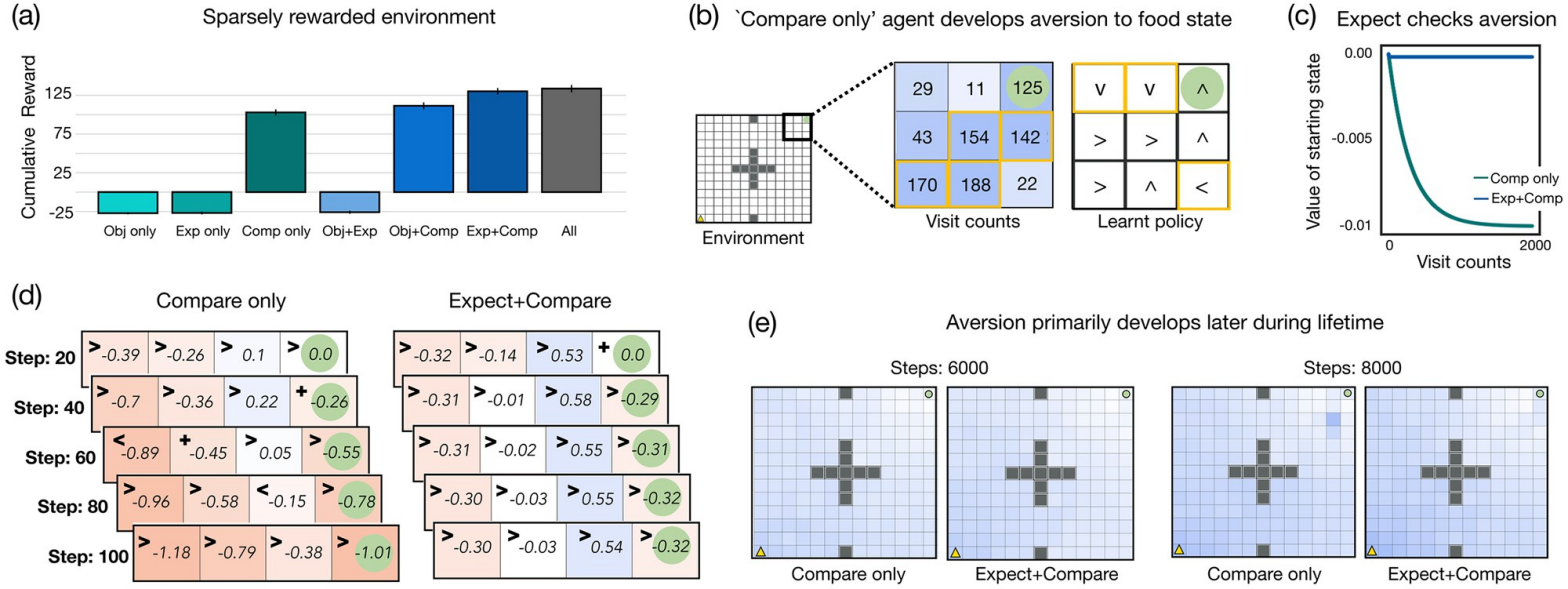
Relative comparisons can lead to undesirable behavior in sparsely rewarded environments. **(a)** Mean cumulative objective reward attained by the agents in a distribution of 13 × 13, stationary environments requiring lifetime learning (lifetime = 12500 steps). While the ‘Compare only’ agent performs relatively well, it is significantly outperformed by the ‘Expect+Compare’ and the ‘All’ agent. **(b)** Visualization of the visit counts and the learnt policy of the ‘Compare only’ agent for states near to the food state. The agent does not visit the food state as often as it visits some of the nearby non-rewarding states (highlighted in yellow). The agent’s learnt policy suggests that it has developed a form of aversion to the food state as it takes a needlessly long route to reach the food state. **(c)** Graph showing how the value of the starting state (which provides an objective reward of 0) changes as a function of the visit counts for the ‘Compare only’ and ‘Expect+Compare’ agents. As the ‘Compare only’ agent keeps re-visiting the starting state, it keeps assigning a lower value to this state (due to its aspiration level). On the other hand, due to prior expectations, the ‘Expect+Compare’ agent prevents this value from becoming too negative. **(d)** Development and prevention of aversion in the simple 4-state environment (the agent is teleported to *s*_1_ after reaching food state). Each interaction shows the agent’s current estimation of the best action to take at each state and its estimated *Q*-value of taking that action at that state. Here, the aspiration level of the agents is deliberately set to be very high. The ‘Compare only’ agent develops an aversion to the food state (at step = 60 and = 80) whereas the ‘Expect+Compare’ agent does not exhibit any aversion behavior. **(e)** Visualization of the visit counts of the ‘Compare only’ and the ‘Expect+Compare’ agent (darker shade represents greater visit counts and vice-versa). At the 6000^*th*^ timestep, the visit counts of the two agents are comparable. At the 8000^*th*^ timestep, the ‘Compare only’ develops aversions and visits states near to the food state more often than it visits the food state.

As shown in Fig 4a, the ‘Objective only’ agent (*w*_1_ = 0.9), ‘Expect only’ agent (*w*_2_ = 0.2), and the ‘Objective+Expect’ agent (*w*_1_ = 0.1, *w*_2_ = 0.6) perform very poorly and attain low cumulative objective reward in their lifetime (*α* = 0.5, *ϵ* = 0.1 for all three agents). This is not surprising as reinforcement learning (and exploration more generally) in sparsely rewarding environments is known to be a challenging problem [22, 44, 48, 57, 58]. The ‘Compare only’ agent (*w*_3_ = 0.1, *ρ* = 0.001, *α* = 0.5, *ϵ* = 0.1), performs relatively well (*M* = 102.86, *SD* = 39.91) and obtains higher cumulative objective reward than the ‘Objective only’, ‘Expect only’, and the ‘Objective+Expect’ agent. The addition of the Expect component to the ‘Compare only’ agent is very helpful as we find that both the ‘Expect+Compare’ (*M* = 130.14, *SD* = 43.49; *w*_2_ = 0.6, *w*_3_ = 0.1, *ρ* = 0.01, *α* = 0.7, *ϵ* = 0.1) and the ‘All’ agent (*M* = 133.58, *SD* = 48.21; *w*_1_ = 0.9, *w*_2_ = 0.9, *w*_3_ = 0.3, *ρ* = 0.01, *α* = 0.7, *ϵ* = 0.1) obtain the highest cumulative objective reward and perform better than the ‘Compare only’ agent.

To understand why the Compare component is not sufficient by itself to maximize cumulative objective reward, we construct a simplified version of the previous gridworld environment—we remove all poison and sinkhole states and instead of being teleported to a random state in the world, the agent is always teleported back to the starting state whenever it reaches the food state. The ‘Compare-only’ agent (*M* = 132.16, *SD* = 15.51) again performs worse than the ‘Expect+Compare’ (*M* = 145.15, *SD* = 11.13) as well as the ‘All’ agent (*M* = 152.65, *SD* = 14.06) in this setting. Fig 4b shows one instance of the visit counts and the learnt policy of the ‘Compare-only’ agent at the end of its lifetime in this environment. The agent visits the states next to the food state quite often but it does not visit the food state as much as it visits these nearby non-rewarding states. The learnt policy of the agent is even more surprising—the agent learns a policy that encourages it to visit the food state but this policy is rather sub-optimal as the agent takes an unnecessarily long route to reach the food state. For example, if the agent is at the state which is to the immediate left of the food state, then following the learnt policy, the agent would take 3 steps to reach the food state from that location (whereas the optimal policy would reach the food state in just 1 step, by taking the action ‘right’). One explanation for this behavior is that perhaps the ‘Compare only’ agent develops some form of ‘aversion’ to the food state. Whenever the agent visits the food state, it teleports back to the starting state location. The starting state locations give a negative subjective reward to the agent (since *ρ* = 0.001) and the agent quickly assigns a negative value to these states. Once the agent starts visiting the food location more frequently, it also inadvertently visits the starting states (due to teleportation) and keeps assigning an even lower value to these states (they provide a subjective reward = −0.001 at each visit). Thus, the agent eventually starts avoiding the food state in order to avoid going back and re-visiting the highly negatively valued starting states. The ‘Expect+Compare’ agent does not develop an aversion to the food state because the Expect component ensures that the value estimate of the starting state locations does not become too negative—whenever the Compare component reduces the value of the starting state, the Expect component induces a positive subjective reward (since it expects a negative reward at the state but instead receives an objective reward equal to 0). This is also shown in Fig 4c which plots the state value estimate of the starting state location as a function of the visit count for the two agents (see also the S1 Text for a derivation of the convergence and the difference in the upper and lower bound for the starting states for the two agents).

We provide an illustration of how aversion can develop and how the Expect component helps the Compare component using the 4-state environment. The agent’s starting location is *s*_1_ and it is always teleported back to this location whenever it visits the food location at *s*_4_. To simulate aversion, we deliberately set the aspiration level of the agents to be 0.6, considerably higher than the optimal aspiration level (= 0.2). Fig 4d shows a visualization of one instance of the agents’ interaction history with the environment. For each interaction, we show the agent’s current policy i.e., the estimated best action to take at each state (where ‘<’ corresponds to taking the action ‘left’, ‘>’ corresponds to taking the action ‘right’, and ‘+’ corresponds to taking the action ‘stay’) and the estimated value of taking that action at that state. In the beginning, at step = 20, the learnt policy of the ‘Compare only’ agent is optimal and it takes the correct action at each state. At step = 40, the agent still follows the optimal policy but the value of taking the action ‘right’ at states *s*_1_ and *s*_2_ is highly negative (and much lower than what it was at step = 20). At step = 60, the agent’s policy becomes sub-optimal and it estimates the best action to take at *s*_1_ to be ‘left’ and the best action to take at *s*_2_ to be ‘stay’. Eventually, at step = 100, the agent learns to take the correct actions at all the states and its policy becomes optimal again. The ‘Expect+Compare’ agent does not exhibit any aversion behavior and the learnt policy of the agent remains optimal throughout its lifetime. These simulations highlight an important drawback of the ‘Compare only’ agent—when comparisons are left unchecked, it can lead to too much pessimism and the agent might end up learning a sub-optimal policy especially in settings where rewards are sparsely available.

#### The importance of dynamic aspiration levels

Until now, we have considered ‘Compare only’ agents that have a fixed aspiration level throughout their lifetime. While having a fixed aspiration level can help the agent to learn in a variety of densely rewarded settings, it fails to perform as well in the sparse reward setting. In a sparse reward environment, the fixed aspiration level can be useful in the initial stages as it helps the agent to find the food location relatively quickly. However, after a certain amount of time, when the agent starts to visit the food state constantly, the same aspiration becomes too high for optimal learning. Fig 4e plots one instance of the visit counts of the ‘Compare only’ and the ‘Expect+Compare’ agent at the 6000^*th*^ timestep as well as the 8000^*th*^ timestep. At the 6000^*th*^ timestep, the two agents are comparable to each other and there is very little difference in the visit count plots. At the 8000^*th*^ timestep, the ‘Compare-only’ agent starts to show the aversion behavior and visits states near to the food state more often than it visits the food state suggesting that aversion develops primarily during the later stages of its lifetime.

While one way to address this shortcoming is to include the Expect component, another way is to have a *dynamic* aspiration level i.e., an aspiration level that changes during the agent’s lifetime. We find that for the ‘Compare only’ agent, an optimal strategy is to start with the optimal aspiration level (i.e., the fixed value of the aspiration level which resulted in the highest cumulative objective reward) and then lower the aspiration level by 10% midway during the agent’s lifetime. Starting off with the optimal aspiration level allows efficient exploration of the environment in the beginning of the lifetime. The lowered aspiration level then ensures that the value of the non-food states does not become too negative and the agent does not develop any aversion to the food state. This strategy considerably improves the agent—the comparison-based agent with the dynamic aspiration (*M* = 145.15, *SD* = 11.13) performs better than the ‘Compare only’ agent with the fixed aspiration level as well as the ‘Expect+Compare’ agent. An important point to note is that the above strategy of setting the dynamic aspiration level is specific to this particular environment—just like a reward function, the aspiration level also needs to reflect the statistics of the environment. Here, the right strategy was to lower the aspiration level after a certain amount of time and some settings might require a different strategy altogether.

#### Interim discussion

The above simulations suggest that relative comparison is a potent reward signal and prior expectation serves as a useful aid to this reward function.

In Experiment 1a, we found that relative comparisons encourage the agent to avoid going to the non-rewarding states it has already visited and instead incentivize the agent to explore novel states. In other words, relative comparisons speed up learning by providing an *exploration incentive* to the agent. Research in reinforcement learning has also suggested various strategies to encourage exploration by providing appropriately structured intrinsic rewards to an agent [43, 44, 59, 60]. For example, curiosity-based methods motivate exploration by providing rewards based on how the agents’ prediction errors change over time and allow an agent even to operate entirely in the absence of objective rewards. While curiosity-based strategies are efficient in domains where rewards barely exist, they can be ineffective in environments where some rewards are available since they do not make optimal use of that particularly critical information, and instead overspend time working out how to control facets of the environment that may not be important. On the other hand, comparison sharpens the information available from rewards and provides a more educated exploration guidance to the agent (when the aspiration level is set properly). Thus, we view the exploration incentive provided by relative comparisons as being complementary to such curiosity-based methods; it is quite likely that biological agents might use comparisons along with signals such as curiosity to guide their exploration in complex environments. In S1 Text, we also provide an assessment of relative comparison against optimistic initialization of the Q-values, finding that the former cannot be reduced to the latter.

In Experiment 1b, we found that prior expectation and relative comparison make the agent more robust to fluctuations and changes in an environment. First, expectation and comparison-based agents do not assign a very high value to the rewarding state—in a way, they prevent the agent from becoming too happy with what it has. Then, when the reward changes its location, they issue a high negative subjective reward which implicitly encourages the agent to explore other states in the environment, allowing it to discover the new reward location quickly. Finally, simulations in sparsely rewarded environments (Experiment 1c) revealed an important shortcoming of relative comparisons—although comparison still speeds up learning in this setting, the agent can become overly pessimistic and learns a sub-optimal policy. Here, the addition of prior expectation helps as it keeps the pessimism in check by signalling to the agent that things are not bad as they seem. We also found that having a dynamic aspiration level improves performance and helps overcome this shortcoming. This emphasizes the importance of having well-tuned aspiration levels to ensure optimal performance of comparison-based agents.

### Experiment 2: Exploring dynamic aspiration via multi-armed bandits

In the previous section, we showed that while relative comparisons provide a valuable exploration incentive, it is crucial to keep their aspiration level in check to prevent sub-optimal learning. We also saw that having a fixed aspiration level can be sometimes detrimental to the agent and it instead needs to have an aspiration that changes within its lifetime. In this section, we conduct simulations in simpler bandit tasks, to gain a deeper understanding of dynamic aspiration levels.

#### Environment design

For this set of experiments, we conduct simulations using the multi-armed bandit environment. In the bandit task, at each timestep, the agent chooses one of the *K* options (‘arms’), and receives a reward that depends stochastically on the chosen arm. The goal of the agent is to maximize its total reward. Unlike the previous gridworld environment, the multi-armed bandit task has no state transitions. Hence, the reward obtained depends only on the chosen action and the learning agent has to explore the action set in order to find the best arm. The multi-armed bandit problem represents one of the simplest RL problems and and is widely employed in psychology and neuroscience for studying decision-making under uncertainty [61–63]. We use this environment because it provides a controlled setting in which to simulate and study aspiration levels, and also allows us to replicate our previous findings (in the gridworld environment) using a well-established paradigm.

We begin by simulating stationary bandits where the reward distribution remains constant over time (Experiment 2a). We use a 10-armed bandit environment where the best arm always pays an objective reward drawn from a Gaussian distribution with mean 1 and standard deviation 1. The remaining 9 arms pay an objective reward according to Gaussian distributions with mean *μ*_*a*_ < 1 for arm *a* and a standard deviation of 1. We modulate task difficulty by manipulating how close (or distant) the best arm is to the other remaining arms. Specifically, we consider two settings. In the first setting, the arms are evenly distributed such that the mean of each of the 9 suboptimal arms is drawn from a uniform distribution on the range [−1, 0.9]. In the second setting, the arms are very close to each other with the means of the arms are drawn from a uniform distribution on the range [0.87, 0.97].

As in the previous experiment, we also simulate non-stationary bandits where the reward distributions change over time (Experiment 2b). We consider two different scenarios. In the first scenario, the reward distribution changes suddenly within the agent’s lifetime and the agent needs to adapt to the new condition in order to perform well. In the second scenario, the reward distribution changes constantly over time (with the reward mean of the arms following a random walk) and hence, the agent needs to continuously adapt throughout its lifetime.

Since our primary goal in this experiment is to understand dynamic aspiration, we here compare the following reward functions: ‘Objective’, ‘Fixed compare’, and ‘Dynamic compare’. ‘Objective’ captures a reward function that is based only on the absolute reward value of the selected arm. ‘Fixed compare’ compares the reward value of the arm to an aspiration level which remains fixed through the agent’s lifetime. In contrast, ‘Dynamic compare’ compares the reward value to an aspiration level that can change within the agent’s lifetime. As before, we assume that the aspiration level is provided to the agent by the designer and we perform grid search to find the optimal aspiration for both ‘Fixed compare’ and ‘Dynamic compare’ agents (see Methods). For comparison, we also include the Upper Confidence Bound (UCB) algorithm [64, 65], which has known performance guarantees and is normatively well-motivated [66, 67]. The UCB algorithm defines a trade-off between an arm’s current expected value and associated uncertainty and chooses the arm with the highest upper confidence bound of the mean (see Methods for mathematical details). Note that the ‘Expect’ agent performs very similarly to the ‘Objective’ agent in the MAB experiments and hence we do not include the Expect component for the following simulations.

#### Exp 2a: Stationary multi-arm bandit task

We first simulate a 10-armed bandit task where the mean of the 9 suboptimal arms is drawn from a uniform distribution on range [−1, 0.9]. To evaluate how well the reward functions fulfill the designer’s objective, we compare the average cumulative objective reward at the end of the agents’ lifetime of 5000 steps.

**Fig 5:**
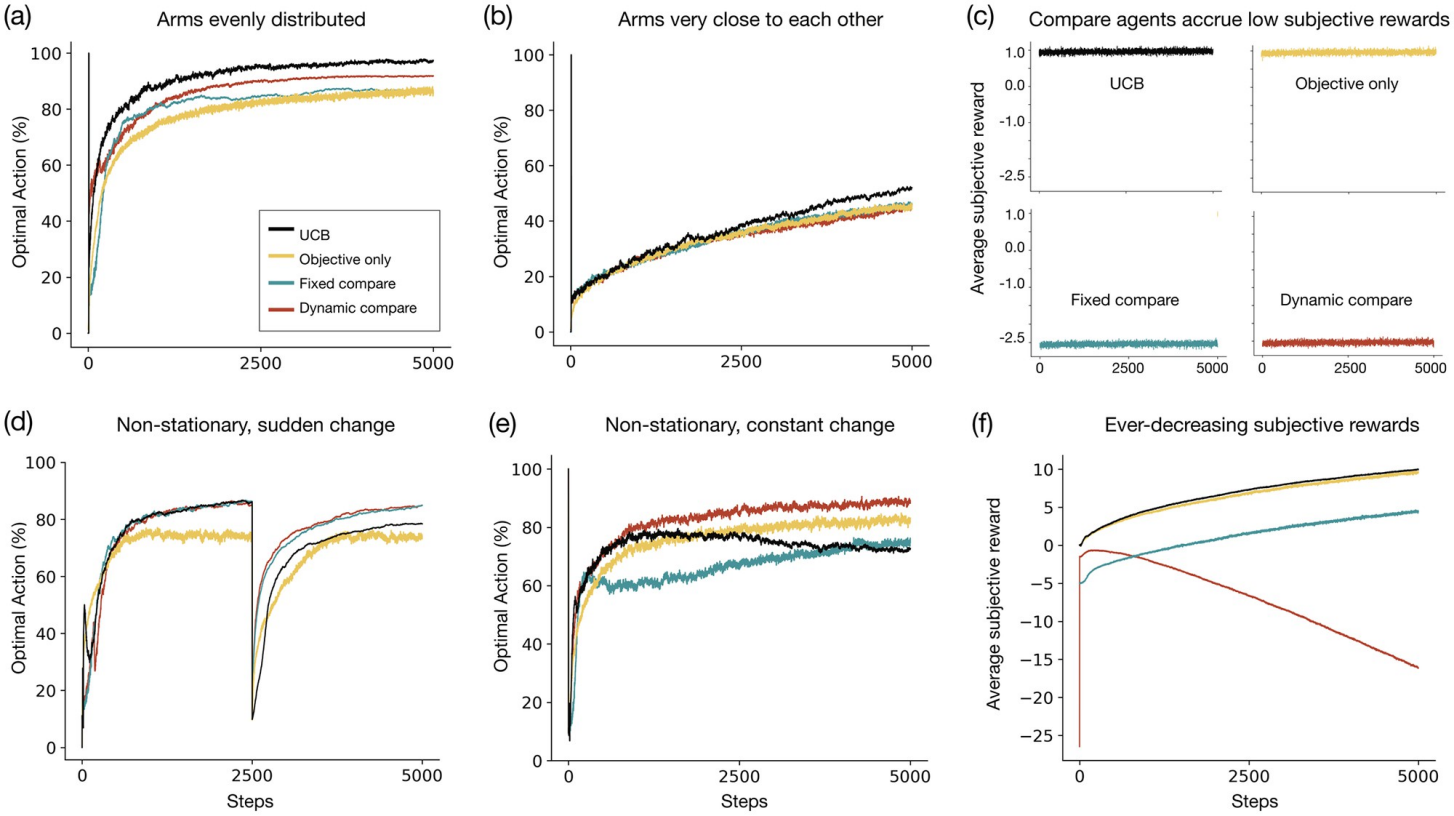
Results of the multi-armed bandit experiments. **(a)** 10-armed bandit simulation where the mean of the 9 sub-optimal arms is drawn from a uniform distribution on range [−1, 0.9]. The graph plots how frequently the agents select the best arm in their lifetimes. The ‘Fixed compare’ agent learns faster than the ‘Objective’ agent and selects the optimal action at a higher rate, especially early during training. The ‘Dynamic compare’ agent selects the optimal action at a higher rate throughout its lifetime compared to these two agents. **(b)** Bandit task where the arms are very close to each other. Here, the comparison-based agents and the ‘Objective only’ agent select the optimal action at a similar rate throughout their lifetime (and the UCB selects the optimal action at a higher rate). **(c)** Plot of the average subjective reward of the agents in the previous bandit task. Compared to the ‘Objective only’ and the UCB agent, the comparison-based agents experience lower subjective rewards (due to their aspiration level). This seems needless since comparisons do not help the agents make better choices. **(d)** Non-stationary bandit task where the reward distribution changes abruptly during the agent’s lifetime. Compared to the ‘Objective only’ agent, the comparison-based agents select the optimal action at a higher frequency, especially after step = 2500 i.e., when the environment changes. **(e)** Non-stationary bandit task where the reward distribution changes constantly during the agent’s lifetime. Early during training, the ‘Fixed Compare’ agent selects the optimal action at a relatively good rate but it is then comfortably outperformed by the other agents. The rising aspirations of the ‘Dynamic Compare’ agent allows it adapt to the changes in the environment and it selects the optimal action at a very high rate throughout the lifetime. **(f)** Despite accumulating high objective rewards, the subjective rewards experienced by the ‘Dynamic Compare’ agent keep decreasing due to its constantly increasing aspiration.

We find that the ‘Fixed compare’ agent (*ϵ* = 0, *ρ* = 3.5) obtains higher cumulative objective reward (*M* = 4648.57, *SD* = 182.93) than the ‘Objective’ agent (*M* = 4384.07, *SD* = 219.66;*ϵ* = 0.1). In some sense, this replicates our findings in Experiment 1a as the gridworld environment requiring *one-time* learning is slightly similar to the stationary bandit task (in both settings, the agent needs to explore various states/actions and then has to exploit the best option). Next, our grid search (see Methods) reveals that the best dynamic aspiration strategy is to increase the aspiration gradually up to a certain point (*ϵ* = 0, strategy: *ρ* = 2 from step 0–50, = 2.5 from step 50–100, = 2.75 from step 100–150, = 3 from step 150–200, = 3.5 afterwards) and the ‘Dynamic compare’ agent which uses this strategy attains a higher cumulative objective reward (*M* = 4796.07, *SD* = 326.45) than the ‘Fixed compare’ agent and the ‘Objective’ agent. Lastly, as expected, the UCB algorithm achieves the highest cumulative reward (*M* = 4875.8066, *SD* = 170.81) and outperforms all other agents. Fig 5a plots how frequently the agents select the optimal action (i.e., the best arm) in their lifetimes. The ‘Fixed compare’ agent learns faster than the ‘Objective’ agent and selects the optimal action at a higher percentage early during training. By the end of their lifetime, both the agents select the optimal action at a similar rate. The ‘Dynamic compare’ agent selects the optimal action at a higher rate at most points during its lifetime implying that it learns a more efficient policy.

We next simulate a 10-armed bandit task where the arms are very close to each other. Here, all arms provide a high objective reward and the best arm is not very distinct from the other arms. In this setting, comparison provides no additional benefit—the cumulative objective reward obtained by both ‘Fixed compare’ (*ϵ* = 0.1, *ρ* = 3.5; *M* = 4786.41, *SD* = 133.16) and ‘Dynamic compare’ (*ϵ* = 0.1, *ρ* = 2.5 from step 0–50 and *ρ* = 3.5 after; *M* = 4790.73, *SD* = 132.74) is similar to that of ‘Objective’ agent (*ϵ* = 0.1; *M* = 4792.05, *SD* = 134.63). Comparison also does not help the agent make better choices—there is no difference between how frequently the comparison-based agents and the ‘Objective’ agent select the best arm in their lifetimes (Fig 5b). At the same time, as shown in Fig 5c, the comparison-based agents constantly receive lower subjective rewards (due to their aspiration). If we again assume that the happiness of an agent is proportional to the subjective reward value, then here the comparison-based agents can be considered as being very unhappy in their lifetimes. While the comparison agents were also more unhappy in the previous bandit task, here this misery seems unnecessary as comparison does not help the agent perform better. In sum, in an environment with many similar options, the comparison-based agents suffer from the ‘paradox of choice’ [36] as comparison does not result in any improvement in performance and only leads to dissatisfaction.

#### Exp 2b: Non-stationary multi-arm bandit task

We now study aspiration levels using non-stationary bandits. We first simulate ‘sudden change’ where the reward distribution of the arms changes abruptly during the agent’s lifetime. We use the 10-armed evenly distributed environment from the previous simulation, where the mean of the 9 sub-optimal arms is drawn from a uniform distribution on range [−1, 0.9]. We simulate non-stationarity by randomly shuffling the reward distribution of the arms midway during the agent’s lifetime (e.g., arm 1 could end up having the reward distribution of arm 10 and vice-versa). Replicating our findings in Experiment 1b, we find that the ‘Fixed compare’ agent (*ϵ* = 0, *α* = 0.1, *ρ* = 3.5) obtains higher cumulative objective reward (*M* = 4561.3, *SD* = 235.11) compared to the ‘Objective’ agent (*ϵ* = 0.1, *α* = 0.1; *M* = 4191.19, *SD* = 180.47) (also see Fig 5d). Next, our grid search reveals that having a dynamic aspiration is not necessary in this setting as the ‘Fixed compare’ agent accumulates a similar cumulative objective reward to the best ‘Dynamic compare’ agent (*M* = 4558.55, *SD* = 240.11) and also selects the best action at a similar rate in its lifetime (Fig 5d). Note that the difference in the pattern of the choices between Fig 5a and 5d for the first 2500 steps is due to the different learning rule used in the non-stationary environment (see Methods). We also note that after the environment changes (i.e. after step = 2500), the comparison-based agents outperform the UCB algorithm which is unsurprising since the standard UCB algorithm is not designed to handle non-stationarity.

We next simulate ‘constant change’ where the reward distribution of the arms changes constantly throughout the agent’s lifetime. We use a 10-armed bandit where the reward mean of the arms follows a random walk. At time *t* = 0, the rewards of the 10 arms are drawn from a Gaussian distribution with mean 0 and standard deviation 1. Then, at each time step, we change the mean reward of the arms by adding a normally distributed increment with mean 0 and standard deviation 0.1. In this environment, having a fixed aspiration level is quite harmful as the ‘Fixed compare’ agent (*ϵ* = 0.1, *α* = 0.1, *ρ* = 5) obtains the lowest cumulative objective reward (*M* = 30879.62, *SD* = 9933.37), lower than the ‘Objective’ agent (*ϵ* = 0.1, *α* = 0.8;*M* = 32220.31, *SD* = 9930.40). Next, using grid search, we find that the best dynamic aspiration strategy is to start with a moderate aspiration (*ρ* = 1.5) and then increase the aspiration constantly throughout the lifetime (by 0.005 at every time step, also see Methods) and the ‘Dynamic compare’ agent which uses this strategy achieves the highest cumulative objective reward (*ϵ* = 0, *α* = 0.1; *M* = 34609.12, *SD* = 10717.57), greater than all other agents. We note that the dynamic aspiration strategy applied here has a different form from the one that worked best in the gridworld environment (Exp 1c), where it was beneficial to lower the aspiration level after a certain point. This is because in that environment, a high aspiration level was only useful in the early stages of training (as it encouraged exploration) but it later became sub-optimal due to the constant teleportation. Here, because the reward distribution of the arms changes constantly (which means that a current sub-optimal arm may become optimal in the future), an ever-increasing aspiration level allows continual exploration and learning, at least over the timecourse of the experiment. Fig 5e shows that early during training, the ‘Fixed compare’ agent selects the optimal action at a relatively high rate but then this aspiration becomes sub-optimal very quickly. The ‘Dynamic compare’ agent keeps adapting to the changes in the environment and selects the optimal action at a higher rate at all times. However, the constantly rising aspiration of the ‘Dynamic compare’ agent has some unintended consequences—the agent receives lower subjective rewards over time (also see Fig 5f). Thus, despite accumulating higher objective rewards over time, internally, the agent experiences constant dissatisfaction due to its ever-increasing aspiration. In some sense, the agent is showing a dissociation between ‘liking’ and ‘wanting’, where it keeps pursing new goals despite not liking what it receives [68–70]. In summary, in constantly changing environments, having a fixed aspiration level is detrimental and results in the agent being overtaken and left behind. At the same time, while constantly rising aspiration help in achieving better performance, they also result in ever-increasing dissatisfaction.

## Discussion

Sensibly or not, people often find it hard to remain happy with what they have. One enjoys a newly bought car for a time, but over time it brings fewer positive feelings and one eventually begins dreaming of the next rewarding thing to pursue. As a consequence, we keep getting lured by the promise of unfathomable future happiness whilst hardly enjoying the riches of the present. Here, we have presented a series of simulations that suggest that these seemingly maladaptive “flaws” might perhaps play an important role in promoting adaptive behavior. Using the idea of reward design, we explored the value of adaptive expectations and relative comparisons as a useful reward signal and found that across a wide range of environments, these features help an agent learn faster and be more robust to changes in the environment. Thus, even though comparisons to the past and future often induce unhappiness, they might still have a beneficial pervasive influence on cognition as they can motivate one to escape the unpleasant or (even worse) mundane present.

In the remainder of the paper, we connect our findings to psychological research on happiness, consider potential shortcomings of our work, and discuss implications of our results for disorders such as depression and overconsumption.

### Connections to psychological research on happiness

Although our setup is limited in many respects, our findings have some potential connections and implications to research on human happiness and well-being.

#### Affluence and happiness

In identifying situations where comparisons can be harmful, our results provide insights to some modern day problems related to affluence and well-being. For instance, we found that when an agent is faced with many similar options, comparisons resulted in constant dissatisfaction without any improvement in performance (Exp 2a). Thus, one lesson that can be taken from our results is that when presented with many similar choices, a decision-maker is better off curtailing comparisons and making decisions without relying on them. This accords with the view that given the explosion of choices in modern times, learning to accept good enough will increase satisfaction and simplify decision-making [36, 71].

We also found that in continually changing environments (Exp 2b), constantly rising aspiration helped in achieving better performance but also resulted in ever-increasing dissatisfaction. This is in line with research that suggests that affluence often only has weakly positive, or even negative, correlation with subjective well-being, primarily because an improvement in conditions leads to a rise in aspiration which subsequently dampens happiness and satisfaction [72, 73]. At the same time, we also saw in Exp 2b having a fixed aspiration level can be quite harmful and results in sub-optimal performance. This relates to the literature on the ‘aspiration-poverty trap’—individuals with low social and economic background often have lower aspirations than rich individuals, which can lead to underachievement and lower levels of investment thereby perpetuating a negative cycle (e.g., a large amount of high-achieving, low-income students do not apply to any selective college or university despite the fact that these selective institutions would often cost them less) [74–77]. Thus, a potential message of these findings is that one should have high aspirations in order to get out of unpleasant situations, but it is then important eventually to curtail them when one is finally in favorable circumstances. Of course, this leaves an open question about how a decision-maker can come to manage and curtail comparisons in the first place. Indeed, an extensive literature in positive psychology has developed various interventions such as practicing gratitude, counting good things, and following mindfulness to enhance well-being and happiness [78–81]. An interesting future direction would be studying the computational mechanisms underlying such interventions as well as investigating possible methods via which an agent can set and learn its own aspiration level. A promising direction in this vein could be studying how aspiration levels might be shaped via a functional relationship between a model-free and model-based system [82, 83]. For instance, a model-based system might alter the aspiration level of the model-free system based on fluctuations in the environment. This in turn could also be helpful to understand what leads someone to develop unreasonably high aspirations [84–86].

#### Optimal levels of happiness

Our findings provide computational support to a growing body of psychological research which documents the “dark side” of being too happy and are consistent with early philosophical ideas that extreme levels of any emotion, including happiness, can be undesirable [87–90]. We observed that an agent’s internal happiness was not necessarily reflective of their performance in the environment and both being too happy and too unhappy led to unwanted outcomes. Agents with unreasonably high aspiration or expectations developed sub-optimal behavior and were also very unhappy in their lifetimes (due to unmet aspirations and expectations). Similarly, agents that had very low aspiration or expectations also performed poorly as they were prone to getting stuck at local minima. However, these agents, despite accumulating very low objective rewards, were very happy in their lifetimes. More broadly, our simulations suggest an inherent trade-off between happiness and performance—in most environments, agents that obtain the highest objective rewards tend to be moderately unhappy in their lifetimes. This is also loosely comparable to the finding that people who experience slightly lower levels of happiness are more successful in terms of income and education level compared to people with the highest levels of happiness [91]. In general, one implication of our work is that it might be *optimal* to have agents that are not always happy and are instead caught in a cycle of never-ending wants and desires. That said, it is worth stating that our simulations do not consider the possibility that an agent can intervene on its own happiness. For instance, in certain settings, a meta-controller could influence an agent to maintain high levels of happiness once it reaches a desired performance threshold (which would allow the agent to be successful and also be happy). In this vein, an interesting direction for future research could be studying the possible role of meta-cognition in influencing happiness.

#### Evolutionary pressures and happiness

Our results also speak to a literature in economics that explores the types of evolutionary pressures that could have produced habituation and relative consumption [92–95]. Similar to our work, these studies model happiness using the metaphorical principal-agent framework, where the principal (evolution) wishes the agent to be maximally fit and has the ability to choose the utility function of the agent to her best advantage. One such study shows that when an agent has limited ability to make fine distinctions (i.e., it cannot tell apart two values that are within a small distance from each other) and when it has a limited range of utility levels (i.e., it has a bound on the minimum and maximum level of happiness it can experience), then evolution would favor a utility function that is adaptive and depends on relative comparisons [95]. In our view, the primary contribution of these studies is showing how cognitive limitations could have favored a happiness function that depends on prior expectations and relative comparisons, and our work complements these studies by suggesting that, regardless of the agent’s constraints, this function could have also been favored because of the learning advantages it confers.

### Relation to mood and anhedonia

Closely related to our research is recent work that posits a role for mood in learning [96–99]. In these proposals, mood is formalized as the moving average of reward prediction errors (and more recently, an estimate of the Advantage function [96]), and is considered to represent environmental momentum. Momentum indicates whether an environment is improving or worsening and can be an important variable for adaptive behavior. Our results augment these studies by showing how (myopic) reward prediction errors (in the form of prior expectations) are a valuable aid to relative comparisons and accelerate learning in a wide variety of environments. Studying the interaction of mood with prior expectations and relative comparisons is an important question for future work.

One observation in the context of mood is that the sorts of adaptive relativities for learning that we have discussed can lead to instabilities in evaluation—modeling aspects of dynamic diseases, such as bipolar disorder [97]. Certainly, the subjective values of states that are taught by the subjective reward functions can vary greatly from their objective values, which is problematic if, for instance, the parameters of the subjective reward function change over time. More generally, given that aspiration levels are an interesting lever for the brain to pull on itself, examining its consequences for psychiatric dysfunction in a range of environments [100, 101] is an important direction for future research. It would also be worth exploring whether dysfunctions such as anhedonic depression [102–104] partly arise because of problems with subjective rather than objective components of reward sensitivity. The same issues might be more broadly relevant, given the chain of reasoning that leads from disturbed average rates of reward [105] to altered motivation in depression [106], potentially negative symptoms in schizophrenia [107], and indeed transdiagonistically across a number of psychiatric and neurological conditions [108]. Nevertheless, it would be remiss not to point out the careful distinctions made between hedonic and motivational aspects of rewards, as between ‘liking’ and ‘wanting’ [53, 54], that we have blurred.

### Limitations

Our work has several limitations which should be addressed in order to draw more concrete parallels between our simulation-based results and psychological research on happiness. For one, we assumed that the agent designer directly provided the reward function to the agent and the agent had no say in what reward function it received. This simplification meant that we were not able to study how an agent might develop biased expectations or aspirations as well as study the consequences of an agent being able to control its own happiness. A productive avenue for future research could be studying reward design using the meta-learning framework, such that an agent learns to choose the parameters of its happiness function in response to the environment it faces [109, 110]. Relatedly, we also did not investigate in detail the potential interaction of discounting with prior expectations and relative comparisons (since we kept a fixed value for the discount factor in our experiments). Studying this further would be an important question for the future. Another limitation of our work is that we did not consider how aspirations can be influenced by social comparisons. Future research could address this by conducting multi-agent simulations wherein agents also compare themselves to other agents in the environment. This could also help understand how relative comparisons might interact with other components of happiness such as guilt and jealousy. Future work should also consider how the components of happiness we have considered here might interact with other affective states such as anxiety [111] and boredom [112]. Lastly, while our choice of environments was driven in part due to their popularity within the RL community, it is not completely clear how much our results will generalize to more real-world situations and therefore, caution must be exercised when generalizing our simulation results.

### Concluding remarks

We conclude by providing some perspective on the problem of overconsumption, an extremely pressing issue that severely threatens future generations. Constant habituation to modern luxuries and ever-rising aspirations are leading us to consume Earth’s natural resources at an alarming rate and resulting in rapid deterioration of our planet [113–117]. Paradoxically, people in modern societies are hardly more satisfied than previous generations [118–121], yet we keep becoming caught in the rat race of consumption and continuing the modern obsession of growth at all costs [122–126]. One implication of our results is that given how advantageous habituation and relative comparisons are in promoting adaptive behavior, it could be possible that these features might be very deeply entrenched in our minds. Thus, any steps to reduce overconsumption will also need serious considerations on how to tackle these biases of the human mind and will require the expertise of scientists from multiple disciplines. For better or worse, we are prone to becoming trapped in a cycle of never-ending wants and desires, and it is more urgent than ever to develop concrete policies and large-scale interventions to reduce habituation and comparisons.

## Methods

### Experiment 1

#### Implementation details

To derive the optimal reward function, we performed a dense grid search over the weights *w*_*i*_, from 0 to 1 with increments of 0.1; this resulted in the consideration of 1330 different reward functions (since there are 11 reward functions between the interval [0, 1] and 11 × 11 × 11 = 1331; then subtract 1 to exclude the reward function *w*_1_ = *w*_2_ = *w*_3_ = 0). Further, since we assume that the agent designer directly provides the aspiration level to the agent, we also performed a dense grid search over the aspiration level, from 0.001 to 1 with increments of 0.05. To ensure that any difference in the performance of the agents was primarily due to the difference in their reward function and not due to a sub-optimal rate of learning or a poor *ϵ*-greedy strategy, we (approximately) optimized the learning rate *α* and the exploration parameter *ϵ* for all the reward functions considered (using a grid search from 0.1 to 0.9 with increments of 0.1). Note that our main results are not significantly affected by the exact value of these parameters (also refer to S1 Text). For the non-stationary environment, we also considered a simple learning rate strategy that adapts to environmental change by tracking the reward prediction error. Specifically, this strategy set the learning rate in proportion to the absolute value of the reward prediction error and thus learned faster when there was a change in the environment. The ‘Objective only’ and ‘Expect+Compare’ agents ended up using this strategy as it helped to improve their performance. Lastly, for all experiments and for all agents, we set the initial *Q*-values to be zero and set the discount factor *γ* equal to 0.99.

#### Evaluation details

The different experiments used different environments (stationary 7 × 7 gridworld in Exp 1a, non-stationary 7 × 7 gridworld in Exp 1b, and stationary 13 × 13 gridworld in Exp 1c). In all experiments, we performed our search of the reward function over a total of 100 environments, where the environments varied in terms of the agent’s starting location, the location of the food, poison, and sinkhole states. We then evaluated how good each reward function was in maximizing the designer’s objective by directly comparing the average cumulative objective reward of each agent over the 100 environments.

In Experiment 1a, in the *one-time* learning environment, we ran each agent for a single episode for a lifetime of 2500 steps. In the *lifetime* learning environment, we ran each agent for a single episode for a lifetime of 12500 steps. In Experiment 1b, we ran each agent for a single episode for a lifetime of 5000 steps. In Experiment 1c, we ran each agent for a single episode for a lifetime of 12500 steps. The lifetime of the agents was decided based on pilot experiments where the criterion was the numbers of steps required by the agents to learn a stable policy.

### Experiment 2

#### Model details

As in Experiment 1, we fix the agents to be *ϵ*-greedy agents where the *Q*-update is performed over the subjective reward function endowed by the agent designer. To study the effect of dynamic aspiration levels, we compared agents with the following reward functions: ‘Objective’, ‘Fixed compare’, and ‘Dynamic compare’. In the stationary environments, upon selecting an arm and receiving reward *r*_*t*_, the agents update the action value of the chosen arm as follows:

$$\begin{array}{c} Q_{t+1}\leftarrow Q_{t}+\frac{1}{k}\left[\left(r_{t}-\rho \right)-Q_{t}\right], \end{array}$$ (11)

where *k* is the number of times the arm has been pulled so far and *ρ* is the aspiration level of the agent. The ‘Objective only’ agent has *ρ* = 0 throughout its lifetime. The ‘Fixed compare’ agent is endowed with a fixed aspiration level at the beginning of each round whereas the aspiration level of the ‘Dynamic compare’ agent can change during training (provided the agent designer changes it). In the non-stationary environments, the agents update the action value of the chosen arm as follows:

$$\begin{array}{c} Q_{t+1}\leftarrow Q_{t}+\alpha \left[\left(r_{t}-\rho \right)-Q_{t}\right], \end{array}$$ (12)

where *α* ∈ [0, 1] is the learning rate. In the stationary environments, the Q-value estimate of an arm is simply the average of the subjective returns received from pulling that arm. In contrast, in non-stationary environments, the agents weight recent pulls more highly than past pulls (if *α* > 0) allowing them to adapt to changes in the environment better. For comparison, we also evaluate the agents against the UCB algorithm [64, 65], which chooses actions deterministically as follows:

$$\begin{array}{c} a_{t}=\underset{k}{\text{argmax}}\;Q_{t}\left(k\right)+\sqrt{\frac{2\;\text{log}\;t}{N_{t}\left(k\right)}}, \end{array}$$ (13)

where *N*_*t*_(*k*) is the number of times action *k* has been selected. Intuitively, at each round, UCB pulls the arm with a good combination of empirical reward estimate and potential benefit associated with the possibility that a relatively undertested arm might be particularly good. The latter factor, associated with the $\sqrt{\frac{2\;\text{log}\;t}{N_{t}\left(k\right)}}$ term helps to avoid always pulling the same arm without considering other arms. This is because as *N*_*t*_(*k*) increases, the chance of pulling that arm decreases.

#### Implementation details

In all simulations, we ran each agent for a lifetime of 5000 steps over 2000 rounds. To ensure that any difference between the agents was primarily caused by their reward functions and not by sub-optimal rate of learning or poor *ϵ*-greedy strategy, we optimized the learning rate *α* and the exploration parameter *ϵ* for all reward functions considered.

For both ‘Fixed compare’ and ‘Dynamic compare’ agents, we derive the optimal aspiration by performing dense grid search. For the ‘Fixed compare’ agent we performed a search from 0.5 to 5 with increments of 0.5 and assigned the aspiration level at the beginning of each round. The aspiration of ‘Dynamic compare’ agent could change during training. For computational tractability, we change the aspiration every 50 steps and derive the dynamic aspiration by performing a grid search from 0.5 to 5 with increments of 0.5 over fixed intervals of 50 steps (i.e., we set a fixed aspiration between steps 0–50 by performing a grid search, and then similarly searched and set a fixed aspiration level between 50–100 steps, and so forth). In the ‘constant change’ simulation (Exp 2b), for the ‘Dynamic compare’ agent, because the environment changes at each step, we changed the aspiration at each step (as opposed to every 50 steps) and again derived the value of this change via grid search (from 0.00 to 0.2 with increments of 0.005).

## Supporting information

S1 Text Supplementary material and results. Includes replication of the results, additional simulation results, value convergence derivation, and additional plots.
(PDF)
