# Supplementary material for: The pursuit of happiness: A reinforcement learning perspective on habituation and comparisons
Source: PLoS Comput Biol. 2022 Aug 4;18(8):e1010316. doi: 10.1371/journal.pcbi.1010316 (PMC9352009; doi:10.1371/journal.pcbi.1010316)
Supplement: S1 Text — Includes replication of the results, additional simulation results, value convergence derivation, and additional plots. (PDF) [file pcbi.1010316.s001.pdf]

The pursuit of happiness: A reinforcement learning perspective on habituation and comparisons

Rachit Dubey [1*], Thomas L. Griffiths[2], Peter Dayan[3,4]

1 Department of Computer Science, Princeton University, Princeton, New Jersey, USA
2 Department of Psychology, Princeton University, Princeton, New Jersey, USA
3 Max Planck Institute for Biological Cybernetics, Tübingen, Germany
4 University of Tübingen, Tübingen, Germany

*Corresponding author: rdubey@princeton.edu

Supplementary Material

Replication of Experiment 1 without poison and sinkhole states

In this section, we replicate our simulations in Experiment 1 using environments that contained no poison and sinkhole states. Apart from the removal of these states, all other experimental details remain the same and we follow the same grid-search method as described in the main text to derive the optimal reward.

Replicating Experiment 1a

We first replicate Experiment 1a by simulating a 7×7 stationary environment that requires one-time learning. Consistent with the findings in the main text (refer to Fig Aa left panel for original data and right panel for replication), we find that the 'Compare only' agent ($w_3 = 0.8$, $\rho = 0.9$) obtains the highest cumulative objective reward, more than the standard reward-based agent, 'Objective only' ($w_1 = 0.3$), as well as the expectation-based agent, 'Expect only' ($w_2 = 0.3$). Further, the 'Compare only' agent outperforms the 'Objective+Expect' agent ($w_1 = 0.9, w_2 = 1.0$), and performs equivalently to the 'Objective+Compare' agent ($w_1 = 0.4, w_3 = 0.4$, $\rho = 0.9$) the 'Objective+Compare' agent ($w_2 = 0.1, w_3 = 0.8$, $\rho = 0.9$), and the 'All' agent ($w_1 = 0.8, w_2 = 0.3, w_3 = 0.9$, $\rho = 0.9$).

Next, we simulate a 7×7 stationary environment that requires lifetime learning. Replicating our results (Fig Ab, left panel shows original data and right panel shows replication), we find that the 'Compare only' agent ($w_3 = 0.5$, $\rho = 0.01$) again accumulates the highest cumulative objective reward greater than the 'Objective only' ($w_1 = 0.8$) and the 'Expect only' agent ($w_2 = 0.1$). Further, the 'Compare only' agent performs equivalently to the 'Objective+Compare' agent ($w_1 = 0.2, w_3 = 0.8$, $\rho = 0.01$), the 'Expect+Compare' agent ($w_2 = 0.1, w_3 = 0.8$, $\rho = 0.01$) as well as the 'All' agent ($w_1 = 0.7, w_2 = 0.1, w_3 = 0.8$, $\rho = 0.01$).

Replicating Experiment 1b

We next simulate a 7×7 non-stationary environment where the food changes its location every 1250 steps. Replicating our results (refer to Fig Ac left panel for original data and right panel for replication), we find that the 'Objective only' agent ($w_1 = 0.5$) performs very poorly, obtaining the lowest objective reward. It is outperformed by both the 'Expect only' ($w_2 = 0.9$) and the 'Compare only' agent ($w_3 = 0.4$, $\rho = 0.9$).

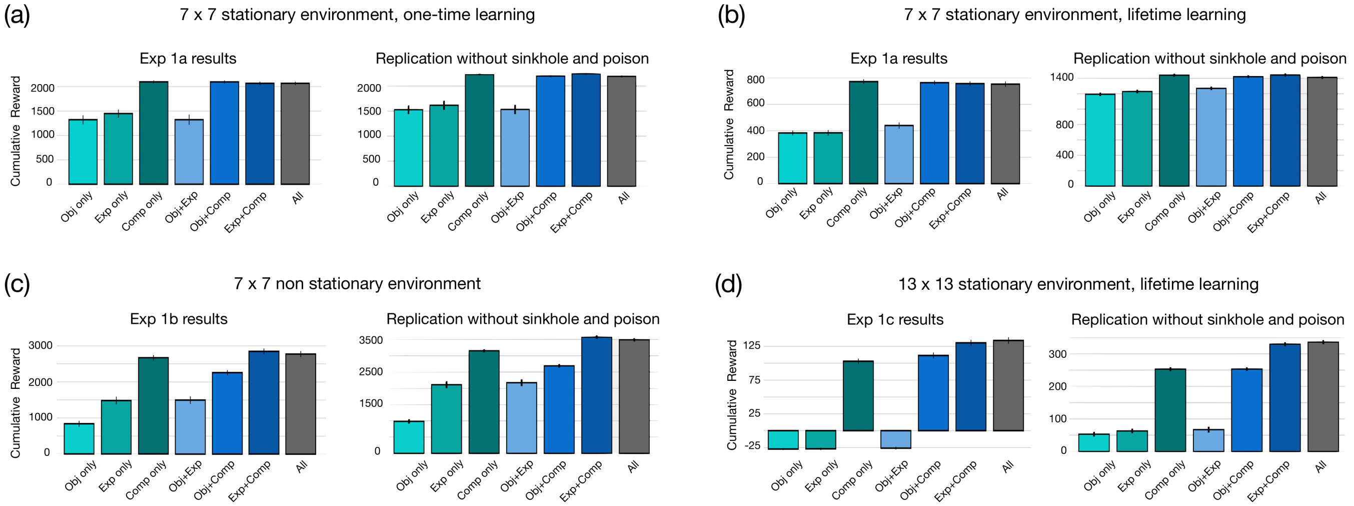

Fig A. Replication of Experiment 1 in environments without poison and sinkhole states (a) Left panel: Data from Exp 1a. Right panel: Mean cumulative objective reward attained by the different agents in a distribution of 7×7 stationary environments requiring one-time learning without any sinkhole and poison (lifetime = 2500 steps). Error bars represent standard error of the mean. **(b)** Left panel: Data from Exp 1a. Right panel: Mean cumulative objective reward attained by the different agents in a distribution of 7×7 stationary environments requiring lifetime learning without any poison and poison (lifetime = 12500 steps). **(c)** Left: Data from Exp 1b. Right: Mean cumulative objective reward attained by the different agents in a distribution of 7×7 non-stationary environments without any sinkhole and poison (lifetime = 5000 steps). **(d)** Left: Data from Exp 1c. Right: Mean cumulative objective reward attained by the different agents in a distribution of 13×13 stationary environments, requiring lifetime learning without sinkhole and poison (lifetime = 12500 steps).

Consistent with our main results, we also see that while the 'Compare only' agent outperforms the 'Expect only' agent, the 'Expect+Compare' agent ($w_2 = 0.1, w_3 = 0.8$, $\rho = 0.9$; mean = 3572.7, std = 394.14) performs considerably better than the 'Compare only' agent (mean = 3151.3, std = 349.79).

Replicating Experiment 1c

We here simulate a 13×13 environment that required lifetime learning (refer to Fig Ad left panel for original data and right panel for replication). Consistent with our main findings, we find that the 'Compare only' agent ($w_3 = 0.1$, $\rho = 0.001$) obtains higher cumulative objective reward than the 'Objective only' ($w_1 = 0.8$), the 'Expect only' ($w_2 = 0.3$) and the 'Objective+Expect' agent ($w_1 = 0.8, w_2 = 0.4$). Again, we find that the addition of the Expect component to the 'Compare only' agent is very helpful as both the 'Expect+Compare' ($w_2 = 0.7, w_3 = 0.1$, $\rho = 0.001$) and the 'All' agent ($w_1 = 0.8, w_2 = 0.9, w_3 = 0.2$, $\rho = 0.001$) obtain the highest cumulative objective reward and perform better than the 'Compare only' agent.

Evaluating relative comparison against optimistic initialization

Experiment 1 showed that a reward function based on relative comparisons speeds up learning by providing an exploration incentive to the agent. In contrast, a reward function that depends on just the objective reward value learns more slowly as it fails to distinguish between novel and previously visited states (if the objective reward is 0 at both the states). One way to incentivize the 'Objective only' agent to explore is

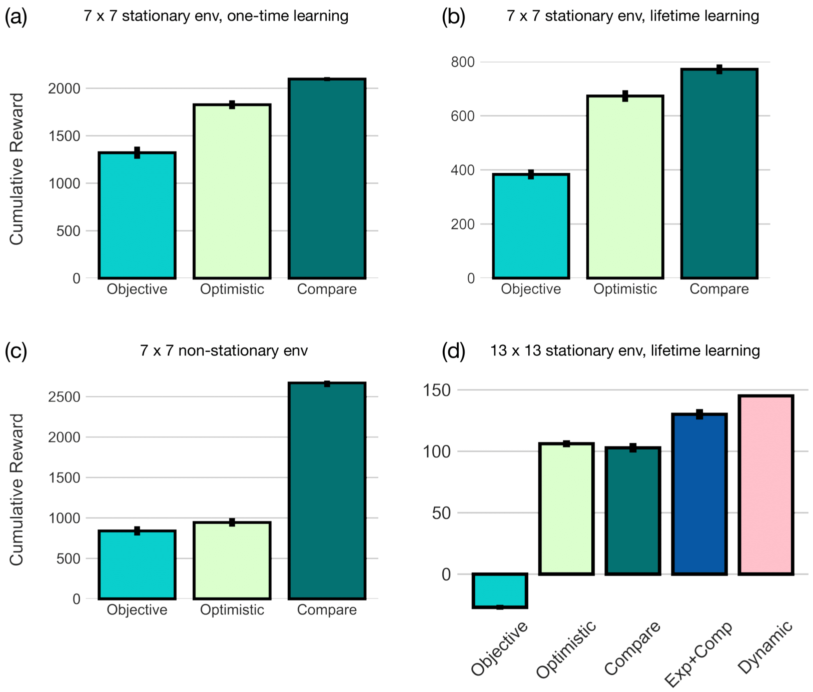

Fig B. Evaluating relative comparison against optimistic initialization (a) Mean cumulative objective reward attained by the 'Objective' (initial Q-values set to 0), 'Optimistic', and 'Compare only' agents in a distribution of 7×7 stationary environments requiring one-time learning. **(b)** Mean cumulative objective reward attained by the three agents in a distribution of 7×7 stationary environments requiring lifetime learning. **(c)** Mean cumulative objective reward attained by the three agents for different in a distribution of 7×7 non-stationary environments. **(d)** Mean cumulative objective reward attained by the different agents in a distribution of 13×13 stationary environments requiring lifetime learning.

to use optimistic initialization such that all the Q-values are assigned initial value larger than zero. This approach assumes that novel state-action pairs are good until proven otherwise, thus encouraging the agent to explore. This is a form of exploration bonus [1, 2, 3] and is also equivalent to potential-based shaping [4, 5]. We here evaluate relative comparisons against optimistic initialization in the simulation environment of Experiment 1. Specifically, we simulate an 'Optimistic' agent, which has the same reward function as the 'Objective only' agent except that we set the initial Q-values as a positive free parameter. As before, we use search to derive an approximately optimal initial Q-values (from 0.1 to 1 with increments of 0.3).

We begin by simulating a 7×7 stationary environment that requires one-time learning (see Fig Ba). As expected, the 'Optimistic' agent ($M = 1826.62, SD = 473.04$; initial Q-values = 1) considerably outperforms the 'Objective' agent (whose initial Q-values are set to zero) thereby demonstrating the value of optimistic initialization. Similarly, the 'Optimistic' agent ($M = 673.12, SD = 215.42$; initial Q-values = 1) also outperforms the 'Objective' agent in a distribution of 7×7 stationary environments requiring lifetime learning (Fig Bb). However, in both these settings, the 'Compare only' agent performs better than the 'Optimistic' agent. This suggests that in dense, stationary environments, the learning and exploration benefits offered by relative comparisons might be greater than that offered by optimistic initialization.

We next simulate a 7×7 non-stationary environment where the food changes its location every 1250 steps (Experiment 1b in the main text). Here, the 'Optimistic' agent ($M = 944.33, SD = 526.77$; initial Q-values = 1) does not perform very well and it is significantly outperformed by the 'Compare only' agent. Thus,

unlike relative comparisons, and as can be expected, optimistic initialization does not help an agent to deal with changes in an environment. This simulation highlights the advantage offered by relative comparisons especially in non-stationary environments which go beyond optimistic initialization.

Finally, we simulate the sparser 13×13 gridworld environment requiring lifetime learning (Experiment 1c in the main text). Here, the 'Optimistic' agent ($M = 106.19, SD = 29.60$; initial Q-values = 1) performs similarly to the 'Compare only' agent possibly because the 'Compare only' agents' performance drops in sparser reward settings due to the development of aversion-like behavior. However, note that the aversion behavior of the 'Compare only' agent can be overcome by either including the Expect component or having dynamic aspiration and both these agents outperform the 'Optimistic' agent. In sum, these simulations show that compared to optimistic initialization, relative comparisons offer some benefits in dense, stationary settings and considerable advantages in non-stationary environments. This suggests that the learning benefits conferred by relative comparisons cannot be reduced to optimistic initialization. An important reason why comparison performs better than optimistic initialization is because the amount of exploration provided by comparisons is continual whereas the amount provided by optimistic initialization is restricted in terms of trials i.e., aspiration-based exploration is forever but optimistic initialization is only during the initial periods.

Simulating the effect of forgetting

The state spaces in our gridworld and the 10-arm bandit tasks are quite large and thus, it might be difficult for a biologically-plausible agent to remember the learned values for all state-action pairs. In this section, we consider possible decay of learned values to study whether our main results might be influenced by forgetting, which can also be seen as an approximately Bayesian counterpart to environmental change.

We first simulate a 10-armed bandit task where the mean of the 9 suboptimal arms is drawn from a uniform distribution on range $[-1, 0.9]$ (Exp 2a in the main text). To simulate forgetting, the action value of each *unchosen* arm is updated as follows:

$$Q_{t+1} \leftarrow (1 - \phi)Q_t, \tag{1}$$

where ϕ ($0 \leq \phi \leq 1$) is the forgetting rate: $\phi = 0$ corresponds to the case without value-decay [6, 7, 8]. The compounding effect of forgetting is such that even a value of ϕ close to 0 results in significant decay during a run. Note that the action value of the chosen arm is still updated as described previously in the main text.

To study the effect of forgetting, we set ϕ equal to 0.02 and find that as expected, forgetting results in poorer performance of both the 'Fixed compare' agent ($\rho = 3.5, \epsilon = 0$) and the 'Objective' agent ($\epsilon = 0.1$). When $\phi = 0$ (as in Exp 2a), the 'Fixed compare' agent obtains higher cumulative objective reward ($M = 4648.57, SD = 182.93$) compared to when $\phi = 0.02$ ($M = 4553.36, SD = 317.34$). Similarly, the 'Objective' agent obtains higher cumulative objective reward when $\phi = 0$ ($M = 4384.07, SD = 219.66$) compared to when $\phi = 0.02$ ($M = 3980.20, SD = 145.16$). Yet, consistent with our main finding in Exp 2a, the 'Fixed compare' agent still outperforms the 'Objective' agent even in presence of forgetting. This is also demonstrated in Fig Ca which shows that the 'Fixed compare' agent selects the optimal action at a higher percentage throughout its lifetime. Note that we do not include 'Dynamic Compare' in the figure as it performs similarly to 'Fixed Compare'. In Fig Cb, we show how the performance of the 'Objective' and 'Fixed compare' agent changes as a function of ϕ and we see that while the performance of the agents drops for higher values of ϕ, the 'Fixed compare' agent still outperforms the 'Objective' agent in all the cases.

We next simulate 'sudden change' where the reward distribution of the arms changes abruptly during the agent's lifetime (Exp 2b in the main text). We set the forgetting rate ϕ equal to 0.02 to study the effect of

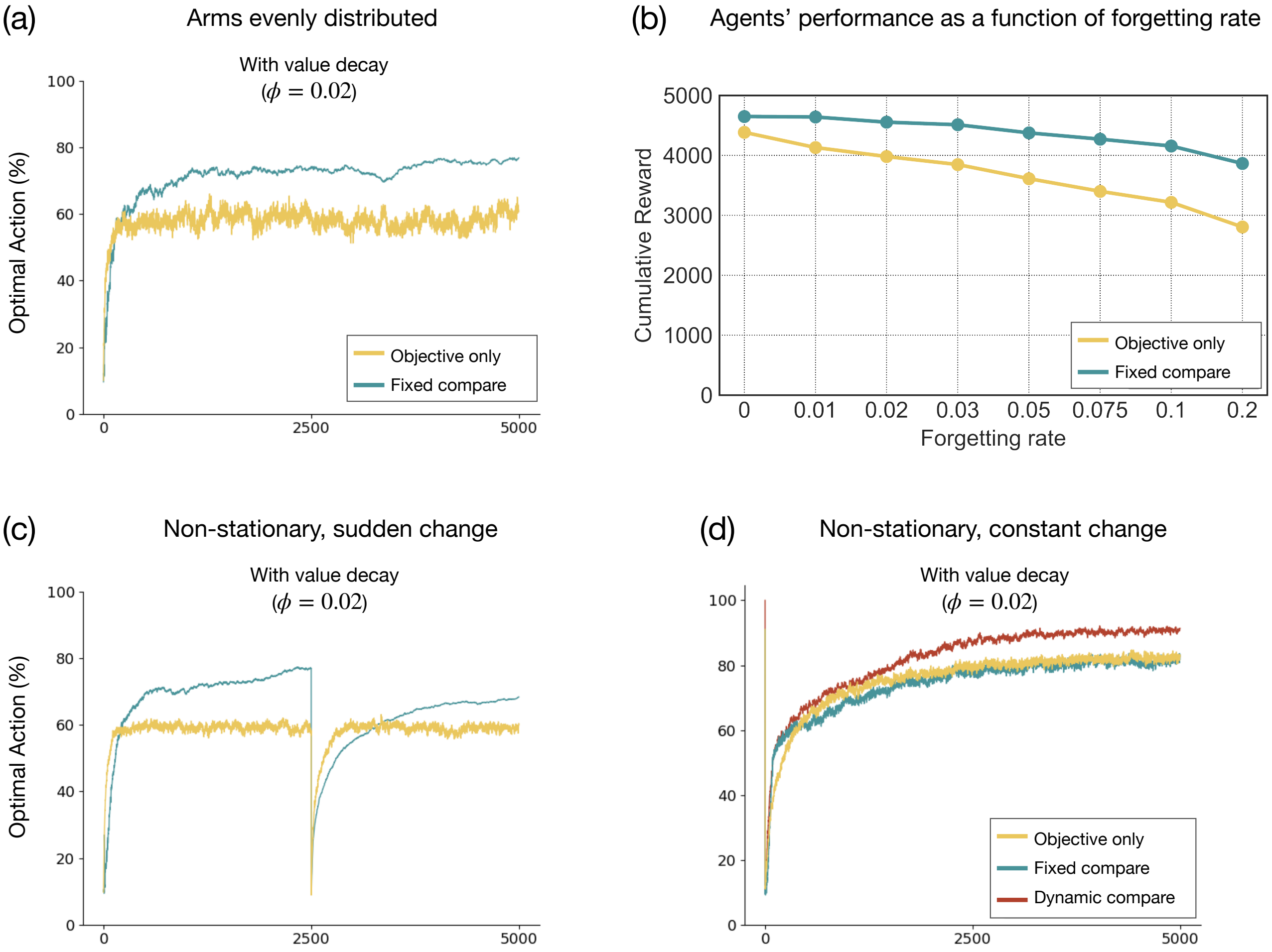

Fig C. Effect of value-decay in the multi-armed bandit tasks (a) Stationary 10-armed bandit task where the mean of the 9 sub-optimal arms is drawn from a uniform distribution on range $[-1, 0.9]$. **(b)** Mean cumulative objective reward attained by the 'Objective' and 'Fixed compare' agent as a function of the forgetting rate. **(c)** Non-stationary 10-armed bandit task where the reward distribution changes abruptly during the agent's lifetime. **(d)** Non-stationary 10-armed bandit task where the reward distribution changes constantly throughout the agent's lifetime.

value decay in non-stationary settings. Value decay again leads to poorer performance of both the 'Fixed compare' ($\rho = 7.5, \epsilon = 0, \alpha = 0.1$) and 'Objective' agents ($\epsilon = 0.1, \alpha = 0.1$). When $\phi = 0$ (as in Exp 2b), the 'Fixed compare' agent obtains higher cumulative objective reward ($M = 4561.3, SD = 235.11$) compared to when $\phi = 0.02$ ($M = 4396.65, SD = 377.40$). Similarly, the 'Objective' agent obtains higher cumulative objective reward when $\phi = 0$ ($M = 4191.19, SD = 180.47$) compared to when $\phi = 0.02$ ($M = 3948.80, SD = 150.89$). Consistent with our main results, the 'Fixed compare' agent still outperforms the 'Objective' agent even in presence of forgetting (see also Fig Cc). Note that 'Dynamic Compare' again performs similarly to 'Fixed Compare' in this setting.

Last, we simulate 'constant change' where the reward distribution of the arms changes constantly throughout the agent's lifetime and again set the forgetting rate ϕ equal to 0.02. Interestingly, in this setting, forgetting improves the performance of the 'Fixed Compare' agent presumably because fixed aspiration still results in continual exploration (due to decay) which can be beneficial in this setting (as sub-optimal

arms may become optimal at some point). When $\phi = 0$ (as in Exp 2b), the 'Fixed compare' agent obtains lower cumulative objective reward ($M = 30879.62, SD = 9933.37$) compared to when $\phi = 0.02$ ($M = 32113.50, SD = 10011.56; \rho = 7.5, \epsilon = 0.1, \alpha = 0.1$). On the other hand, the 'Objective' agent obtains similar cumulative objective reward when $\phi = 0$ ($M = 32220.31, SD = 9930.40$) compared to when $\phi = 0.02$ ($M = 32221.65, SD = 10048.54; \epsilon = 0.1, \alpha = 0.8$). Forgetting also improves the 'Dynamic Compare' agent – when $\phi = 0$, it obtains lower cumulative objective reward ($M = 34609.12, SD = 10717.57$) compared to when $\phi = 0.02$ ($M = 35252.66, SD = 10628.12; \epsilon = 0, \alpha = 0.1, \rho$ increments by 0.01 at every time step). Further, in this setting, the 'Dynamic compare' outperforms the 'Objective' agent and also selects the optimal action at a higher rate (Fig Cd), which is again consistent with our main results. In sum, these simulations lend further evidence to our results and suggest that relative comparisons might still benefit an agent even when there is value decay.

State value convergence

To complement the analysis in Experiment 1b and 1c, we derive the converged value estimate for the food state and the starting state for the different agents.

State value convergence for the food state

We first begin by studying convergence of the value function for the food state. As described in the main text, the learning rule for the agents is given below:

$$
\begin{aligned}
& f = (w_1 + w_2 + w_3).r_t - w_3.\rho - w_2.V(s_t), \\
& \delta = f + \gamma V(s_{t+1}) - V(s_t), \\
& V(s_t) \leftarrow V(s_t) + \alpha\delta.
\end{aligned}
\tag{2}
$$

To study convergence, we assume that the agents are already at the food state and they remain at the food state endlessly (regardless of the chosen action). Thus, the agents receive an objective reward = 1 at every step. Further, since the learning rate α doesn't determine the final converged value estimate (it only determines how slow/fast the value converges), we fix $\alpha = 1$ to ease mathematical computation. Given this, we can compute the value of the food state at any given time step as follows:

$$
\begin{aligned}
& f = (w_1 + w_2 + w_3) - w_3.\rho - w_2.V_t, \\
& \delta = f + \gamma V_t - V_t, \\
& V_{t+1} = V_t + \delta,
\end{aligned}
\tag{3}
$$

where V_{t+1} is the value of the food state at time step $t+1$, and V_t is the value of the food state at time t. Following algebraic manipulations, we can re-write Equation 3 as follows:

$$
V_{t+1} = (\gamma - w_2).V_t + w_1 + w_2 + w_3.(1 - \rho). \tag{4}
$$

Upon solving this recurrence equation, we get:

$$
V_{t+1} = V_0 + \big[w_1 + w_2 + w_3.(1 - \rho)\big].\frac{(\gamma - w_2)^t - 1}{\gamma - w_2 - 1}, \tag{5}
$$

where V_0 is the value of the food state at time step 0 and is simply equal to 0 (as we had set the initial values = 0 for all states at the start of training).

We next compute the value estimate as t approaches infinity:

$$\lim_{t\to\infty} V(t) = \frac{w_1 + w_2 + w_3.(1-\rho)}{\gamma - w_2 - 1}. \lim_{t\to\infty} \big[(\gamma - w_2)^t - 1\big]. \tag{6}$$

In our experiments, we have $\gamma = 0.99$ and $0 <= w_2 <= 1$. Thus, we have $|\gamma - w_2| < 1$. Given this, we have $\lim_{t\to\infty}[(\gamma - w_2)^t] = 0$. Thus, the converged value estimate for the food state is as follows:

$$\lim_{t\to\infty} V(t) = \big[w_1 + w_2 + w_3.(1-\rho)\big].\frac{1}{1-\gamma+w_2}. \tag{7}$$

We now use Equation 7 to derive the converged food state value estimates for the 'Objective only', 'Expect only', 'Compare only', and the 'Expect+Compare' agents.

For the 'Objective only' agent, given $\gamma = 0.99$, the upper bound for the value estimate is 100 (when $w_1 = 1$) and the lower bound for the value estimate is 10 (when $w_1 = 0.1$). Thus, we see that the 'Objective only' agent ends up assigning a very high value to a rewarding state, which can cause problems later when the environment changes (as shown in Exp 1b and Fig 3 in the main text).

For the 'Expect only' agent, the upper bound for the value estimate is 0.9901 (when $w_2 = 1$) and the lower bound for the value estimate is 0.909 (when $w_2 = 0.1$). In some sense, we see that the 'Expect only' agent is very conservative and despite obtaining constant rewards at the food state, it never assigns a very high value to the food state (akin to the hedonic treadmill). This allows it to eventually learn in non-stationary environments (shown in Exp 1b in the main text).

For the 'Compare only' agent, the upper bound for the value estimate is 99.9 (when $w_3 = 1, \rho = 0.001$) and the lower bound for the value estimate is 0.5 (when $w_3 = 0.1, \rho = 0.95$). Thus, if the aspiration of the 'Compare only' agent is sufficiently high, then the agent will not assign a very high value to the food state, which will allow it to learn when the environment changes.

For the 'Expect+Compare' agent, the upper bound is 9.9901 (when $w_2 = 0.1, w_3 = 1, \rho = 0.001$) and the lower bound is = 0.01155 (when $w_2 = 0.1, w_3 = 0.1, \rho = 0.95$). Similar to the 'Compare only' agent, if the aspiration level of the 'Expect+Compare' agent is sufficiently high then it won't assign a very high value to the food state, which will help guide learning in non-stationary environments.

State value convergence for the starting state

We now study convergence of the value function for the starting state. As before, we assume that the agents stay in the starting state endlessly (regardless of the chosen action). Thus, the agents receive an objective reward = 0 at every step. Following the above computation, the converged value estimate for the starting state is as follows:

$$\lim_{t\to\infty} V(t) = -w_3.\rho\frac{1}{1-\gamma+w_2}. \tag{8}$$

From Equation 8, we first see that the 'Objective only' and the 'Expect only' agents always assign a value = 0 to the starting state (since $w_3 = 0$ for these agents). Next, for the 'Compare only' agent, the upper bound for the value estimate is -0.01 (when $w_3 = 0.1, \rho = 0.001$) and the lower bound is -95 (when $w_3 = 1, \rho = -0.95$). Thus, we see that the 'Compare only' agent ends up assigning quite a negative value to the starting state, which can lead to unintended consequences (e.g., it develops aversion to the food state in a sparsely rewarded environment; shown in Exp 1c in the main text).

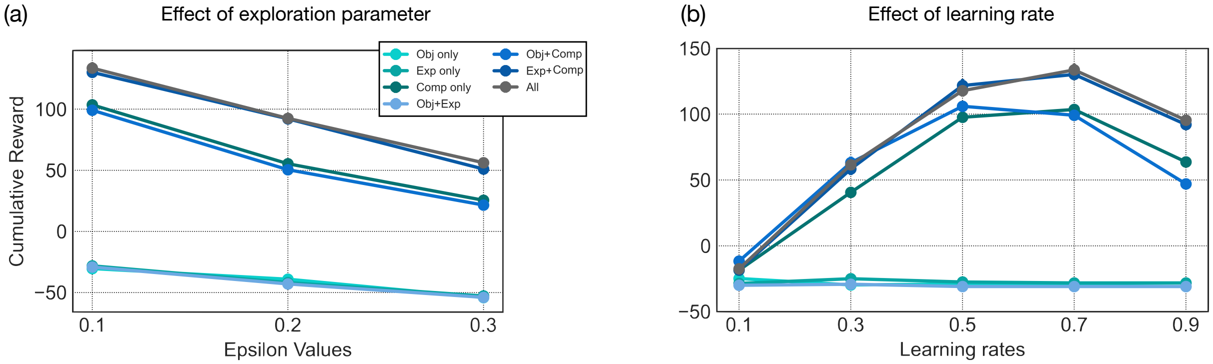

Fig D. Influence of the learning rate and exploration parameters (a) Cumulative objective reward attained by the different agents for different epsilon values in a distribution of 13×13 stationary environments requiring lifetime learning. **(b)** Cumulative objective reward attained by the different agents for different learning rates in a distribution of 13×13 stationary environments requiring lifetime learning.

For the 'Expect+Compare' agent, the upper bound is -9.901 x 10^{-5} (when $w_2 = 1, w_3 = 0.1, \rho = 0.001$) and the lower bound is -8.64 (when $w_2 = 0.1, w_3 = 1, \rho = -0.95$). Thus, we see that the Expect component manages the pessimism induced by the Compare component and it prevents the value of the starting state from becoming extremely negative. This eventually allows the 'Expect+Compare' agent to learn a better policy than the 'Compare only' agent in sparsely rewarded settings.

Influence of the learning rate and exploration parameters

In addition to performing a grid search over the weights w_i and the aspiration level ρ, we also determined an approximately optimal learning rate α and exploration parameter ϵ for all the reward functions considered. That said, our main results are not significantly affected by the exact value of these parameters. As one illustration, Fig D shows how the performance of the different agents changes as a function of different values of ϵ and learning rates for the 13×13 *lifetime* learning environment (Experiment 1c). A low value of ϵ $(= 0.1)$ results in optimal performance for the different agents but the relative performance of the agents remains similar even at larger values. Similarly, while a very low learning rate or very high learning rate results in poorer performance for most agents, the relative performance of the agents remains similar across different learning rate values.

Influence of the different components on the performance of the 'All' agent

In Experiment 1, we suggested that the different components, Objective, Expect, and Compare have different effects on the subjective reward function and the subsequent behavior of the agent. Here, we show how these different components influence the behavior of the 'All' agent in a distribution of 7×7 non-stationary environments (Exp 1b in the main text; recall that here the Objective component hindered the performance of the agent while both prior expectations and relative comparisons improved the performance of the agent).

Fig Ea shows how the performance of the 'All' agent changes as a function of different values of w_1, while fixing w_2 and w_3 at their optimal values ($w_2 = 0.1, w_3 = 0.7$). Evidently, the w_1 i.e. the Objective component is not really necessary for the 'All' agent to perform well as the performance of the agent peaks when $w_1 = 0$ (which is equivalent to the 'Expect+Compare' agent). Further, larger values of w_1 lead to worse performance which shows that the Objective component is deleterious in this setting when the other components are also included.

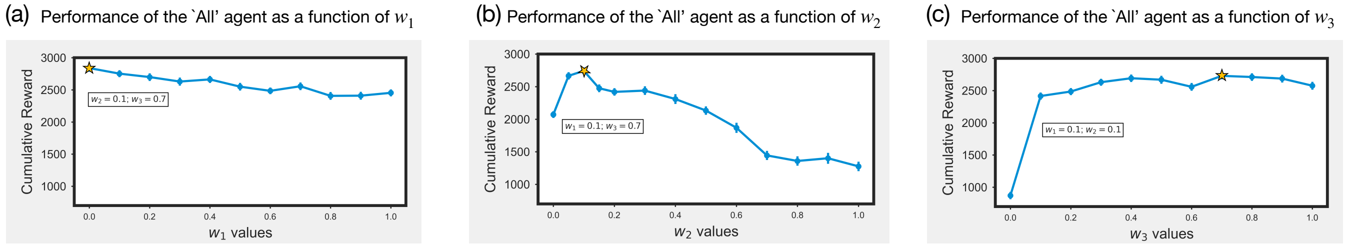

Fig E. Influence of the different components on the performance of the 'All' agent. **(a)** Mean cumulative objective reward accumulated by the 'All' agent in a distribution of 7×7 non-stationary environments for different w_1 values (the optimal w_1 value is marked in yellow). **(b)** Mean cumulative objective reward accumulated by the 'All' agent for different w_2 values (the optimal w_2 value is marked in yellow). **(c)** Mean cumulative objective reward accumulated by the 'All' agent for different w_3 values (optimal w_3 value marked in yellow).

Fig Eb shows how the performance of the 'All' agent changes as a function of different values of w_2, while fixing w_1 and w_3 at their optimal values ($w_1 = 0.1, w_3 = 0.7$). The performance of the agent drops considerably when $w_2 = 0$ and it rises sharply when $w_2 = 0.1$. This implies that the Expect component is a very important component of the 'All' agent. The performance of the 'All' agent also drops sharply as the value of w_2 increases which implies that the optimal performance is only achieved at a certain value of w_2 (when $w_2 = 0.1$) and the exact choice of w_2 is very important in this setting.

Fig Ec shows how the performance of the 'All' agent changes as a function of different values of w_3, while fixing w_1 and w_2 at their optimal values ($w_1 = 0.1, w_2 = 0.1$). Here, the agent performs very poorly when $w_3 = 0$ and its performance rises rapidly as the value of w_3 increases. This suggests that the Compare component is very important for the 'All' agent and optimal performance is achieved at higher values of w_3. Together, this analysis provides evidence on the importance of the Expect component and Compare component in contributing to the performance of the 'All' agent for the non-stationary environment.

Additional simulation plots

This section contains additional simulation plots to complement our main analyses.

Experiment 1b

Fig F, G, H, and I plot the average visit counts and the average learned state values for the 'Objective only', 'Expect only', 'Compare only', and 'Expect+Compare' only agents respectively for the 7×7 non-stationary environment (at the time-steps just before the food location changes).

Experiment 1c

Fig J(a) plots the reward learning curves of the different agents for the 13×13 stationary environment requiring lifetime learning. Fig J(b) provides another visualization of the aversion behavior developed by the 'Compare only' agent, including the learned Q-values at the end of the training (i.e. after 12500 steps).

References

1. Sutton RS. Dyna, an integrated architecture for learning, planning, and reacting. ACM Sigart Bulletin 1991; 2:160–3

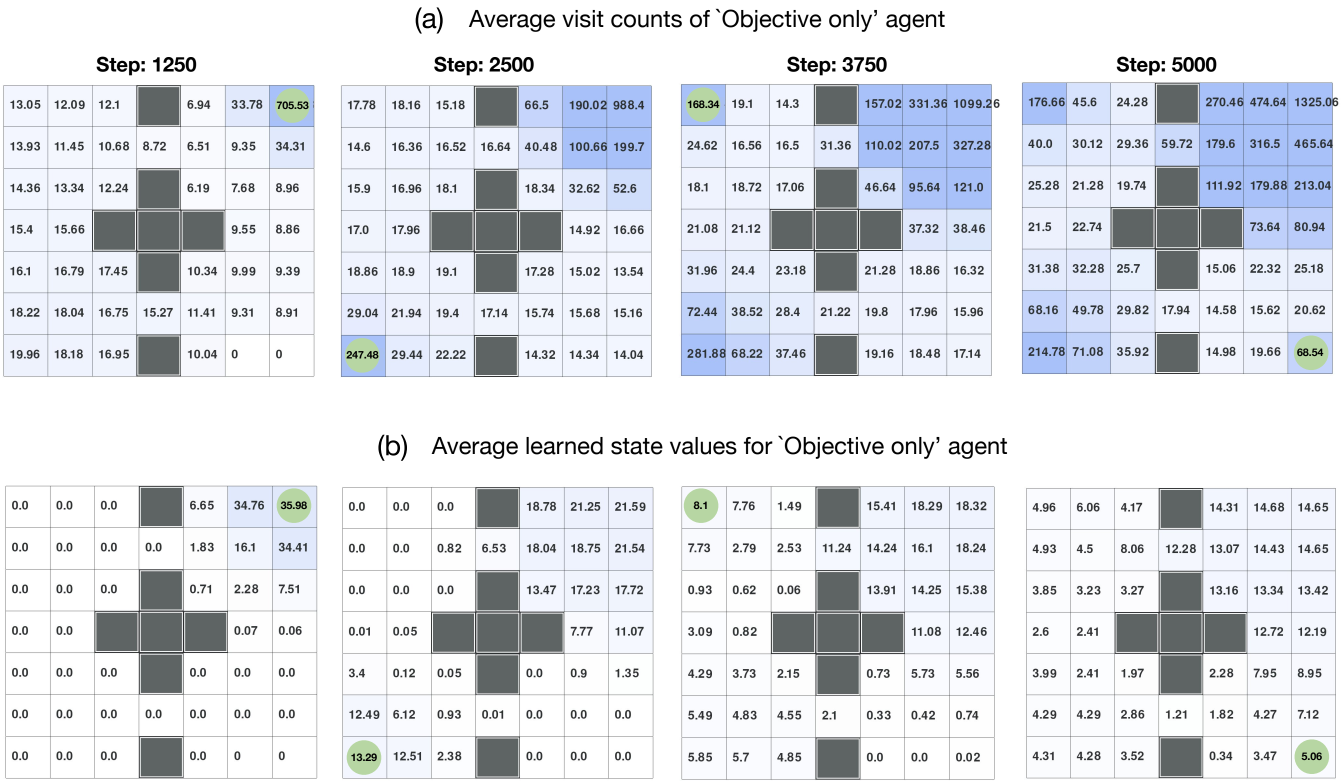

Fig F. (a) Average visit counts of the 'Objective only' agent for the 7×7 non-stationary environment at the time steps just before the food changes location, where darker color represents higher visit counts and vice-versa. **(b)** Average learned state values of the 'Objective only' agent for the 7×7 non-stationary environment just before the food changes location.

2. Dayan P and Sejnowski TJ. Exploration bonuses and dual control. Machine Learning 1996; 25:5–22
3. Kakade S and Dayan P. Dopamine: generalization and bonuses. Neural Networks 2002; 15:549–59
4. Ng AY, Harada D, and Russell S. Policy invariance under reward transformations: Theory and application to reward shaping. *International Conference on Machine Learning*. Vol. 99. 1999 :278–87
5. Wiewiora E. Potential-based shaping and Q-value initialization are equivalent. Journal of Artificial Intelligence Research 2003; 19:205–8
6. Ito M and Doya K. Validation of decision-making models and analysis of decision variables in the rat basal ganglia. Journal of Neuroscience 2009; 29:9861–74
7. Katahira K. The relation between reinforcement learning parameters and the influence of reinforcement history on choice behavior. Journal of Mathematical Psychology 2015; 66:59–69
8. Kato A and Morita K. Forgetting in reinforcement learning links sustained dopamine signals to motivation. PLoS computational biology 2016; 12:e1005145

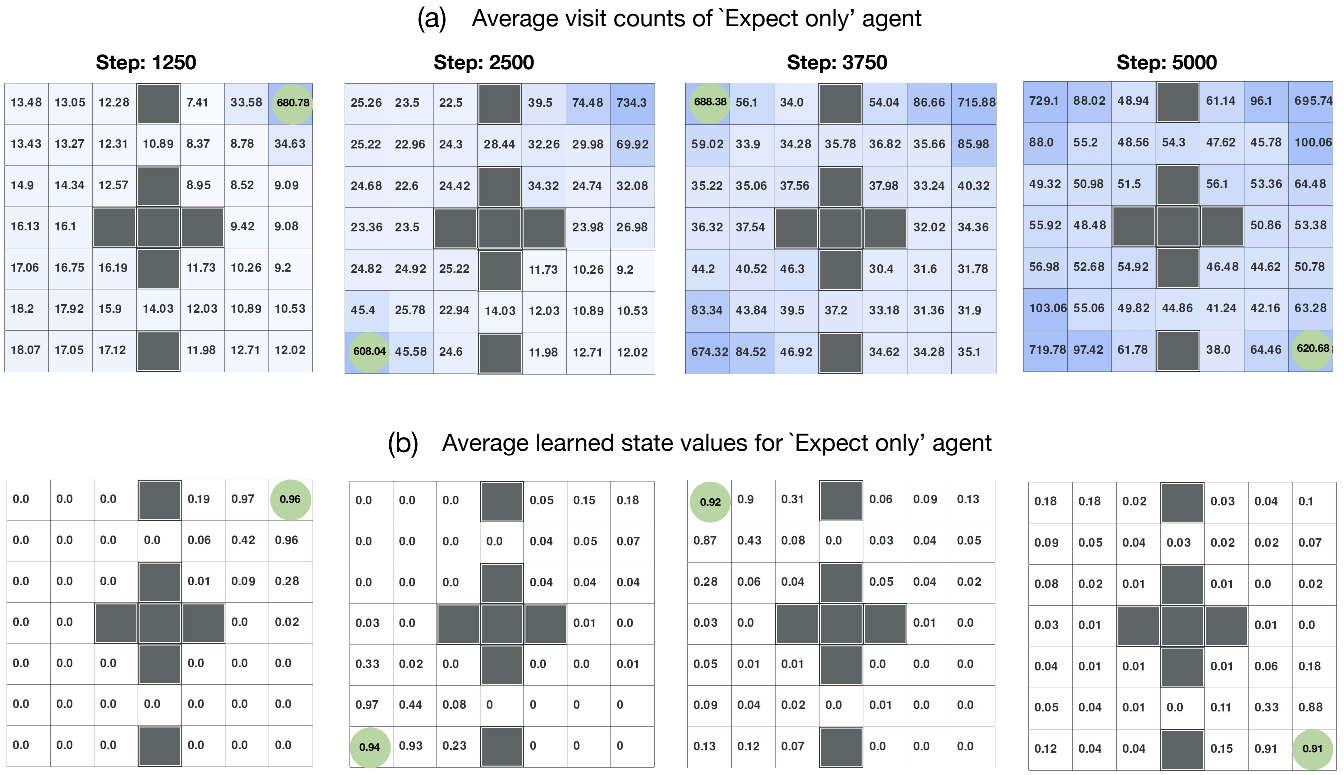

Fig G. (a) Average visit counts of the 'Expect only' agent for the 7×7 non-stationary environment at the time steps just before the food changes location (darker color represents higher visit counts and vice-versa). **(b)** Average learned state values of the 'Expect only' agent for the 7×7 non-stationary environment just before the food changes location.

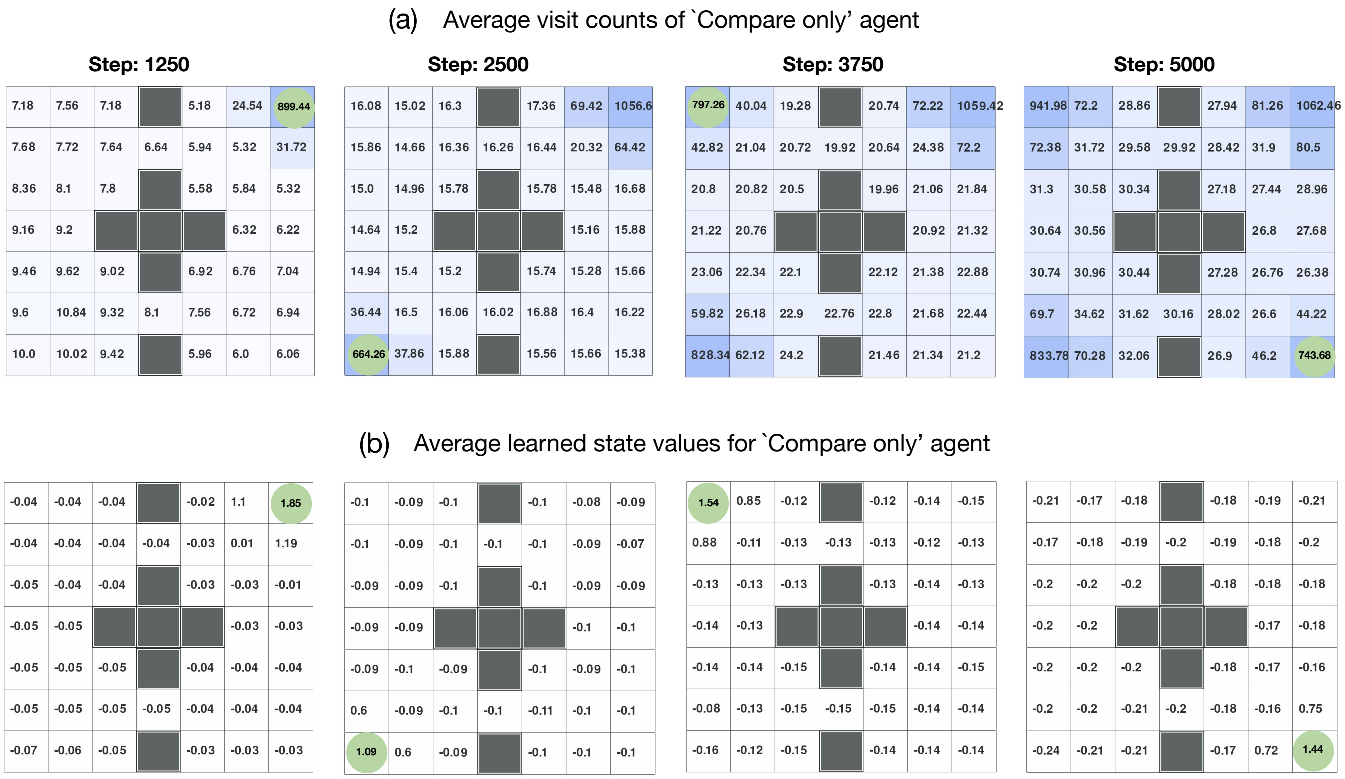

Fig H. **(a)** Average visit counts of the 'Compare only' agent for the 7×7 non-stationary environment at the time steps just before the food changes location. **(b)** Average learned state values of the 'Compare only' agent for the 7×7 non-stationary environment just before the food changes location.

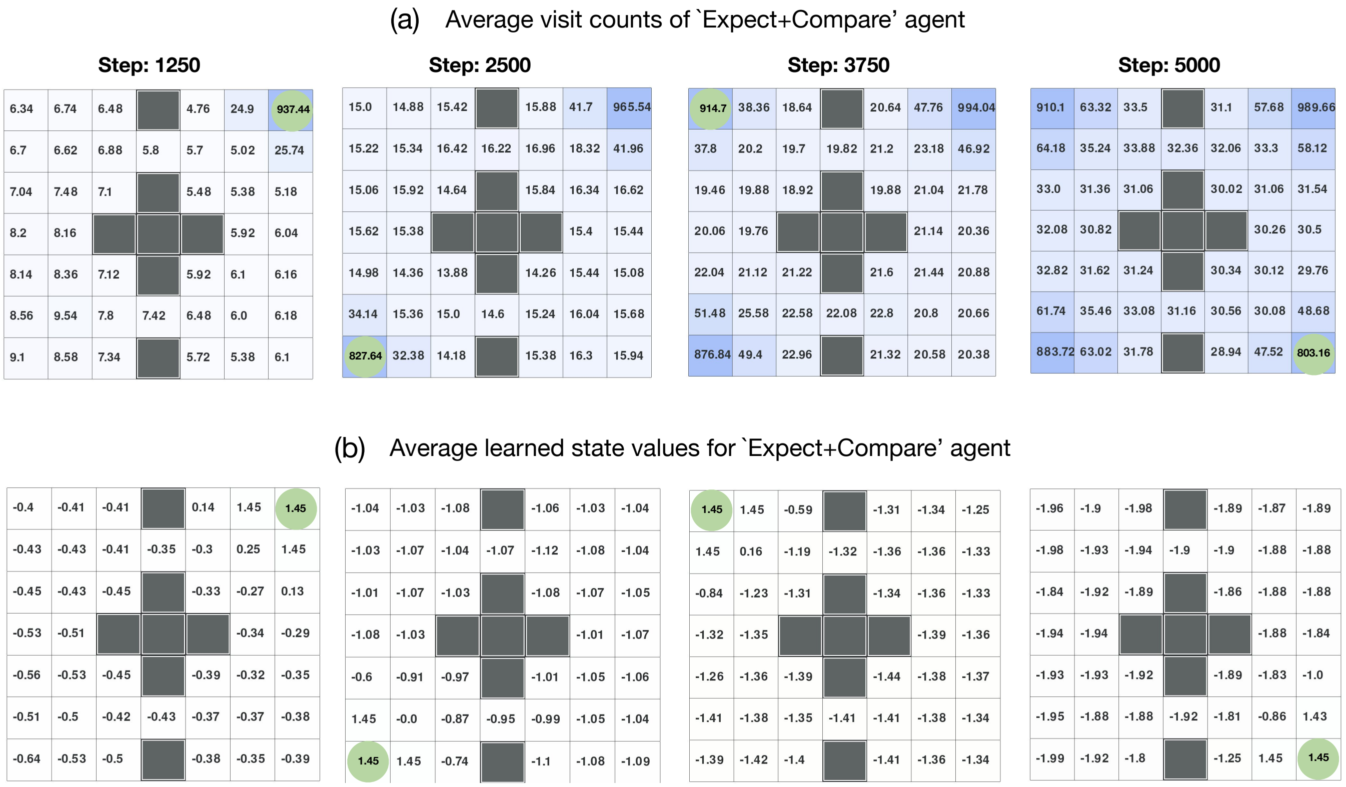

Fig I. (a) Average visit counts of the 'Expect+Compare' agent for the 7×7 non-stationary environment at the time steps just before the food changes location (darker color represents higher visit counts and vice-versa). **(b)** Average learned state values of the 'Expect+Compare' agent for the 7×7 non-stationary environment just before the food changes location.

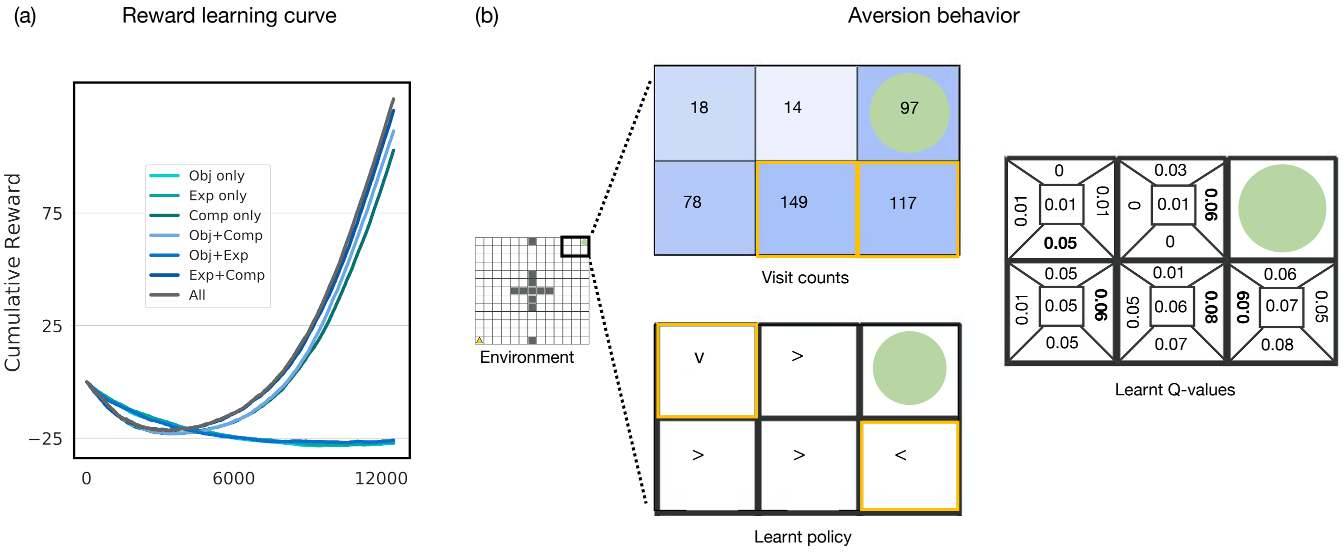

Fig J. (a) Reward learning curves for the different agents for the 13×13 environment requiring lifetime learning. **(b)** Another demonstration of the aversion behavior developed by the 'Compare only' agent in this environment.
